# Hydrogen Peroxide Induced Cell Death: The Major Defences Relative Roles and Consequences in *E. coli*

**DOI:** 10.1371/journal.pone.0159706

**Published:** 2016-08-05

**Authors:** Lionel Uhl, Sam Dukan

**Affiliations:** Institut de Microbiologie de la Méditerranée – Université Aix-Marseille, Laboratoire de Chimie Bactérienne, CNRS UMR7283, 31 Chemin Joseph Aiguier, 13009 Marseille, France; University of the West of England, UNITED KINGDOM

## Abstract

We recently developed a mathematical model for predicting reactive oxygen species (ROS) concentration and macromolecules oxidation *in vivo*. We constructed such a model using *Escherichia coli* as a model organism and a set of ordinary differential equations. In order to evaluate the major defences relative roles against hydrogen peroxide (*H*_2_ *O*_2_), we investigated the relative contributions of the various reactions to the dynamic system and searched for approximate analytical solutions for the explicit expression of changes in *H*_2_ *O*_2_ internal or external concentrations. Although the key actors in cell defence are enzymes and membrane, a detailed analysis shows that their involvement depends on the *H*_2_ *O*_2_ concentration level. Actually, the impact of the membrane upon the *H*_2_ *O*_2_ stress felt by the cell is greater when micromolar *H*_2_ *O*_2_ is present (9-fold less *H*_2_ *O*_2_ in the cell than out of the cell) than when millimolar *H*_2_ *O*_2_ is present (about 2-fold less *H*_2_ *O*_2_ in the cell than out of the cell). The ratio between maximal external *H*_2_ *O*_2_ and internal *H*_2_ *O*_2_ concentration also changes, reducing from 8 to 2 while external *H*_2_ *O*_2_ concentration increases from micromolar to millimolar. This non-linear behaviour mainly occurs because of the switch in the predominant scavenger from Ahp (Alkyl Hydroperoxide Reductase) to Cat (catalase). The phenomenon changes the internal *H*_2_ *O*_2_ maximal concentration, which surprisingly does not depend on cell density. The external *H*_2_ *O*_2_ half-life and the cumulative internal *H*_2_ *O*_2_ exposure do depend upon cell density. Based on these analyses and in order to introduce a concept of dose response relationship for *H*_2_ *O*_2_-induced cell death, we developed the concepts of “maximal internal *H*_2_ *O*_2_ concentration” and “cumulative internal *H*_2_ *O*_2_ concentration” (e.g. the total amount of *H*_2_ *O*_2_). We predict that cumulative internal *H*_2_ *O*_2_ concentration is responsible for the *H*_2_ *O*_2_-mediated death of bacterial cells.

## Introduction

Oxygen is indisputably essential for life, but it can also impair cell ability to function normally or it can participate in its destruction ([1] and [2]) because of the generation of reactive oxygen species (ROS) like hydrogen peroxide (*H*_2_ *O*_2_), superoxide ($O_{2}^{•-}$) or hydroxyl radical (*HO*^•^).

In order to better understand ROS dynamic within cells, we recently developed a mathematical model ([3]) for predicting reactive oxygen species (ROS) concentration and macromolecules oxidation *invivo*. This first study principally focuses on *HO*^•^ dynamic and its consequence on DNA whereas the current study will mainly focus on *H*_2_ *O*_2_ dynamic.

*Escherichia coli* was used as a model organism. In order to build our mathematical model we used data from a large number of articles dealing with enzymes or molecule concentrations (in *E. coli*, kinetic properties and chemical reaction rate constants). We were then able to propose a mathematical model based on a set of ordinary differential equations relating to fundamental principles of mass balance and reaction kinetics. It offers the possibility to simulate properly the experimental results obtained by biologists and therefore to understand the biological parameters involved in the observed phenomena.

The purpose of this study is to use our mathematical model in order to better understand *H*_2_ *O*_2_ mode of action on *E. coli* as a model organism.

In aerobic organisms, oxygen oxidizes redox enzymes, generating a flux of *H*_2_ *O*_2_ that can potentially damage the cell. For instance, *Escherichia coli* generates about 14 *μ*M *H*_2_ *O*_2_ per second when it grows aerobically on glucose ([4]). In order to cope with *H*_2_ *O*_2_ stress, microbes typically contain multiple catalases and/or peroxidases. *E. coli* contains one Alkyl hydroperoxide reductase (Ahp) and two different catalases (Cat). Alkyl hydroperoxide reductase is the primary scavenger for endogenous *H*_2_ *O*_2_ in *E. coli* ([5]). Catalase contributes little when *H*_2_ *O*_2_ levels are low, but it becomes the most effective scavenger when *H*_2_ *O*_2_ levels are high ([5]). Moreover, membrane permeability is part of the global defence process against *H*_2_ *O*_2_ ([4]). However, to our knowledge, the question of their relative involvement remains unsolved especially with regard to the exogenous *H*_2_ *O*_2_ concentration.

Mechanisms involved in *H*_2_ *O*_2_ induced cell death were studied by Imlay and Linn ([6] and [7]) who showed that the exposure of *E. coli* to *H*_2_ *O*_2_ led to two different modes of killing. The first was observed at low *H*_2_ *O*_2_ concentration (1–3 mM *H*_2_ *O*_2_) and resulted from the DNA damage caused by *HO*^•^ ([7]). The second resulted from damage to unknown macromolecules, inflicted more directly, through *H*_2_ *O*_2_-mediated oxidation. However, and to our knowledge, the question of the relative involvement of the cumulative or the maximal *H*_2_ *O*_2_ dose involvement in this phenomenon remains unsolved. Dose response is a question often raised about radiative hazards. For instance Harrison et al. ([8]) indicated median survival times in rats following intravenous injection of polonium-210. The total alpha-particles-emitted numbers show that the cumulative dose and not the maximal dose is principally responsible for death.

Using our mathematical model, we first investigated the relative role of the different ways (principally Ahp, Cat and membrane) for cells to decrease and fight *H*_2_ *O*_2_ oxidative stress. Here we predict that their involvement depends on the *H*_2_ *O*_2_ stress level. Moreover and as observed for radiative hazards, we predict that cumulative internal *H*_2_ *O*_2_ concentration is responsible for the *H*_2_ *O*_2_-mediated death of bacterial cells.

## Materials and Methods

The model assumes that all molecule concentrations are homogeneous in cells. We therefore describe the problem with a dynamic system of ordinary differential equations (ODE) instead of using a complex algorithm such as the Next-Sub-Volume Method. Indeed, one algorithm generally used to study the compartmentalization of molecules in microorganisms (for instance *E. coli*) is the Next-Sub-volume Method. It is a Gillespie-like ([9] and [10]) method approaching the spatial effects of diffusive phenomena and chemical reaction. According to the Next Sub-volume Method, the side length *ℓ* of the square sub-volumes has to satisfy the two inequalities

$$\begin{array}{c} R\ll \ell \;\text{and}\;\tau_{diff}=\frac{\ell^{2}}{6D}⪡\tau_{react}\\ \text{where}\;R\;\text{is the larger radius of a molecule of substrat}\\ \text{and}\;D\;\text{the diffusion constant of}\;H_{2}O_{2}\\ \tau_{diff}\;\text{represents the characteristic time of diffusion}\\ \tau_{react}\;\text{represents the characteristic time of reaction} \end{array}$$

The first inequality indicates that dissociation events can be properly defined within sub-volumes. The second criterion specifies that the time for any molecule to leave a sub-volume is much smaller than the shortest reaction time *τ*_*min*_ among the molecular species, so that all molecules are homogeneously distributed within the sub-volumes. For example, the 3D simulations are typically performed with *ℓ* = 0, 1 *μ*m and the depth *h* = *ℓ* of the sub-volumes, which is many times larger than the average radius of a substrat even protein. Considering the *H*_2_ *O*_2_ molecule maximal number, the reaction initially follows a pseudo-first order kinetic with rate constant *k*′ = *k*[*H*_2_ *O*_2_] and the characteristic time of reaction is therefore *τ* = 1/*k*′. This time has to be compared to the characteristic time of diffusion of *H*_2_ *O*_2_: $\tau_{diff}=\frac{\ell^{2}}{6D}\approx 10^{-6}$ s (with *H*_2_ *O*_2_ diffusion constant *D* = 2 10^−9^ m^2^.s^−1^). This comparison gives *τ* = 1/*k*′ = 1/*k*[*H*_2_ *O*_2_] ≫ 10^−6^ or [*H*_2_ *O*_2_] ≪ 10^6^/*k*. Even with very high rate constant such as 10^6^ M^−1^s^−1^, the inequality imposes [*H*_2_ *O*_2_] ≪ 1 M. In conclusion, while [*H*_2_ *O*_2_] ≪ 1 M, the diffusion within the cell is faster than the reaction rate and we do not need to consider compartmentalization.

$O_{2}^{•-}$ and *H*_2_ *O*_2_ are involved in many reactions. Of course we do not take all possible reactions into account, for instance, we do not consider the Haber-Weiss reaction, because our simulations showed no change with or without its consideration and moreover because the relevance of this reaction *in vivo* is questionable ([11] and [12]); actually adding the Haber-Weiss reaction, numerical simulations show that it is negligible whether *H*_2_ *O*_2_ concentration is under 0.1 mol⋅L^−1^. Using published rate constants, we propose here some simplifications and approximations of the system achieved by neglecting the kinetically non-significant reaction. *HO*^•^ was studied in a previous article ([3]).

### Superoxide kinetics

$O_{2}^{•-}$ is mainly involved in the following kinetically significant reactions:

Its production:

metabolismproduction⟶(k1O2•-

Its dismutation by SOD (superoxide dismutase)

O2•-+H+⟶SOD(k212H2O2+12O2

These two reactions lead to the following ordinary differential equation (ODE) coming from the balance between production and dismutation by SOD:

$$\begin{array}{c} \frac{d[O_{2}^{•-}]}{dt}=k_{1}-k_{2}[SOD][O_{2}^{•-}] \end{array}$$

### Internal hydrogen peroxide kinetics

*H*_2_ *O*_2_ appears significantly in the following reactions: Its productions:

metabolismproduction⟶(k1′H2O2

O2•-+H+⟶SOD(k212H2O2+12O2

Its dismutation by catalase (Cat) or Alkylhydroperoxidase (Ahp)

H2O2⟶(CatH2O+12O2

H2O2⟶(AhpH2O+12O2

Its diffusion across cell membrane

H2O2⟶(kdiffH2O2out

*k*_*diff*_ has been calculated using the membrane permeability coefficient (*P* = 1.6 × 10^−3^ cm/s), the membrane surface area (*A* = 1.41 × 10^−7^ cm^2^) and cell volume (*V* = 3.2 × 10^−15^ L) given by Seaver and Imlay ([4]), therefore $k_{diff}=\frac{P\times S}{V}$.

The ODE becomes:

$$\begin{array}{ccc} \frac{d\left[H_{2}O_{2}\right]}{dt} & = & k_{1}^{\prime }\\ & & +\frac{1}{2}k_{2}\left[SOD\right]\left[O_{2}^{•-}\right]\\ & & -\frac{k_{cat}^{Ahp}\left[Ahp\right]\left[H_{2}O_{2}\right]}{\left[H_{2}O_{2}\right]+K_{M}^{Ahp}}-\frac{k_{cat}^{Kat}\left[Cat\right]\left[H_{2}O_{2}\right]}{\left[H_{2}O_{2}\right]+K_{M}^{Kat}}\\ & & -k_{diff}(\left[H_{2}O_{2}\right]-\left[H_{2}O_{2}\right]{}_{out}) \end{array}$$

where *H*_2_ *O*_2__*out*_ corresponds to *H*_2_ *O*_2_ in the external habitat of the cell.

*K*_*M*_ ($K_{M}^{Kat}$ for catalase and $K_{M}^{Ahp}$ for alkylhydroperoxidase) is the Michaelis constant. *k*_*cat*_ ($k_{cat}^{Kat}$ for catalase and $k_{cat}^{Ahp}$ for alkylhydroperoxidase) is the turnover number, it represents the maximum number of molecules (here *H*_2_ *O*_2_) that an enzyme is able to convert into products per second.

### External hydrogen peroxide

$$\begin{array}{c} \frac{d{\left[H_{2}O_{2}\right]}_{out}}{dt}=k_{diff}\frac{n\cdot V_{in}}{V_{out}-nV_{in}}(\left[H_{2}O_{2}\right]-{\left[H_{2}O_{2}\right]}_{out}) \end{array}$$

The cell density is given by *n*. *V*_*in*_ represents the cell internal volume and *V*_*out*_ corresponds to the total volume. Of course, as microorganisms cannot take up more space than their medium, we have the inequality *V*_*out*_−*nV*_*in*_ ≫ 0.

### Cell density

For under 10 minutes experimental time (consistent with most of our simulation), cell density could be considered as a constant but for long time simulation we propose the logistic equation for cell growing function. The logistic equation (also called the Verhulst model) is a model of population growth first published by Pierre Verhulst ([13] and [14]). The continuous version of the Verhulst model is described by the following differential equation:

$$\begin{array}{c} \frac{dn}{dt}=r\cdot n(1-\frac{n}{n_{max}}) \end{array}$$

where *r* is the Malthusian parameter (rate of population growth) and *n*_*max*_ the maximum sustainable population. This differential equation gives an analytical solution:

$$\begin{array}{c} n\left(t\right)=\frac{n_{0}e^{rt}}{1+\frac{n_{0}}{n_{max}}(e^{rt}-1)} \end{array}$$

where *n*_0_ is the initial density. This value depends on the experiment. We choose carrying capacity *n*_*max*_ = 5 × 10^9^ cell/mL. The maximal rate of growth usually shows that a growing bacterial population doubles at regular intervals near a characteristic time *τ*_*d*_ ≈ 20 minutes. Therefore *n*(*t*) expression also gives:

$$\begin{array}{c} n\left(t\right)=\frac{n_{0}2^{t/\tau_{d}}}{1+\frac{n_{0}}{n_{max}}(2^{t/\tau_{d}}-1)} \end{array}$$

where *r* = ln(2)/*τ*_*d*_.

Nevertheless this characteristic time depends on cell history and stress. For example, even 0.2 mM of *H*_2_ *O*_2_ when added to a logarithmically growing *E. coli* population is enough to generate an immediate decrease in the number of viable cells. This phenomenon is transient and the original number of viable cells is recovered only about 40 minutes after the occurrence of the sub-lethal stress ([15]). This transient phenomenon is mirrored at the population level by a lag phase in which optical density remains almost constant for about 40 minutes. A fraction dies, and then the remaining bacteria resume growth so that the number of viable cells reaches the original number. For instance Chang et al. ([16]) also report a lag phase of about 40 minutes after an addition of 1.5 mM of *H*_2_ *O*_2_. In order to take into account this phenomenon we consider that *τ*_*d*_ → ∞ if *t* < 40 minutes so that *n*(*t* < 40 *min*) = *constant* after *H*_2_ *O*_2_ oxidative stress.

We were not concerned with stationary phase because no experiment carried out in this work reached the maximum sustainable population.

### Kinetic constants

The kinetic constants used in this work are gathered in Table 1 according to Imlay and Fridovich ([17]) and Seaver and Imlay ([5] and [4]).

**Table 1:** Kinetic constants used to describe *H*_2_ *O*_2_ evolution.

Kinetic constants	
*k*_1_	5.7 × 10^−6^ mol⋅L^−1^⋅s^−1^
*k*_2_[*SOD*]	2.8 × 10^4^ s^−1^
*k*_2_	1.5 × 10^9^ mol^−1^⋅L⋅s^−1^
*V*_*in*_	3.2 × 10^−15^ L
$k_{1}^{\prime }$	12 × 10^−6^ mol⋅L^−1^⋅s^−1^
$k_{cat}^{Ahp}\left[Ahp\right]$	6.6 × 10^−4^ mol⋅L^−1^⋅s^−1^
$K_{M}^{Ahp}$	1.2 × 10^−6^ mol⋅L^−1^
$k_{cat}^{Kat}\left[Cat\right]$	4.9 × 10^−1^ mol⋅L^−1^⋅s^−1^
$K_{M}^{Kat}$	5.9 × 10^−3^ mol⋅L^−1^
*k*_*diff*_	70 s^−1^

### Numerical simulations

All numerical simulations were carried out using the MATLAB ODE solver ode15s for stiff differential equations. The multistep solver ode15s is a variable order solver based on the numerical differentiation formulas.

## Results and Discussion

This section presents the analytical study of the dynamic system. This analysis will provide us with insight into the kinetic parameters significantly important for the dynamics of *ROS*.

### Internal hydrogen peroxide

#### Without exogenous stress

In the wild-type strain, $O_{2}^{•-}$ equilibrium is rapidly reached. Indeed the characteristic time of $O_{2}^{•-}$ evolution is 1/*k*_2_[*SOD*] ≈ 35 *μ*s. Therefore we can consider $O_{2}^{•-}$ as a constant and we can assume that $\left[O_{2}^{•-}\right]\left(t\right)\approx {\left[O_{2}^{•-}\right]}_{\infty }$ (S1 File supporting information data for demonstration).

So in terms of changes to internal *H*_2_ *O*_2_ concentration, we approach

k1′+12k2[SOD][O2•-]≈k1′+12k1 because $\left[O_{2}^{•-}\right]\approx {\left[O_{2}^{•-}\right]}_{\infty }$ Let us call k1′+12k1=kprod.

That is a first point, $O_{2}^{•-}$ dismutation by SOD involved nearly an increase of 25% in the endogenous *H*_2_ *O*_2_ production.

Moreover, in the absence of exogenous *H*_2_ *O*_2_, we can consider that:

$$\begin{array}{c} \left[H_{2}O_{2}\right]\ll K_{M}^{Ahp},K_{M}^{Cat} \end{array}$$

so the differential equation system can be simplified to a linear system:

$$\begin{array}{c} \frac{d\left[H_{2}O_{2}\right]}{dt}=k_{prod}-(\frac{k_{cat}^{Ahp}\left[Ahp\right]}{K_{M}^{Ahp}}+\frac{k_{cat}^{Kat}\left[Cat\right]}{K_{M}^{Kat}})\left[H_{2}O_{2}\right]-k_{diff}(\left[H_{2}O_{2}\right]-{\left[H_{2}O_{2}\right]}_{out}) \end{array}$$

$$\begin{array}{c} \frac{d{\left[H_{2}O_{2}\right]}_{out}}{dt}=k_{diff}^{\prime }(\left[H_{2}O_{2}\right]-{\left[H_{2}O_{2}\right]}_{out}) \end{array}$$

with:

$$\begin{array}{c} k_{diff}^{\prime }=k_{diff}\frac{n\cdot V_{in}}{V_{out}-nV_{in}} \end{array}$$

Let us call $\frac{k_{cat}^{Ahp}\left[Ahp\right]}{K_{M}^{Ahp}}+\frac{k_{cat}^{Kat}\left[Cat\right]}{K_{M}^{Kat}}=k_{enz}$, then the differential equation system can be written with a matrix structure:

$$\begin{array}{c} \frac{d}{dt}(\begin{array}{c} \left[H_{2}O_{2}\right]\\ {\left[H_{2}O_{2}\right]}_{out} \end{array})=(\begin{array}{c} k_{prod}\\ 0 \end{array})+(\begin{array}{cc} -(k_{enz}+k_{diff}) & k_{diff}\\ {k^{\prime }}_{diff} & -{k^{\prime }}_{diff} \end{array})(\begin{array}{c} \left[H_{2}O_{2}\right]\\ {\left[H_{2}O_{2}\right]}_{out} \end{array}) \end{array}$$

The matrix eigenvalues are λ_1_ > λ_2_:

$$\begin{array}{c} \lambda_{1}=\frac{-(k_{enz}+k_{diff}+{k^{\prime }}_{diff})+\sqrt{{\left(k_{enz}+k_{diff}+{k^{\prime }}_{diff}\right)}^{2}-4k_{enz}{k^{\prime }}_{diff}}}{2}<0 \end{array}$$

$$\begin{array}{c} \lambda_{2}=\frac{-(k_{enz}+k_{diff}+{k^{\prime }}_{diff})-\sqrt{{\left(k_{enz}+k_{diff}+{k^{\prime }}_{diff}\right)}^{2}-4k_{enz}{k^{\prime }}_{diff}}}{2}<0 \end{array}$$

According to the value of the reaction rate constant, we can make the following approximation: $\lambda_{1}\approx -\frac{k_{enz}}{\left(k_{enz}+k_{diff}\right)}k_{diff}^{\prime }$ and λ_2_ ≈ −(*k*_*enz*_ + *k*_*diff*_).

The full matrix *V* with columns corresponding to the eigenvectors is:

$$\begin{array}{c} V=(\begin{array}{cc} {k^{\prime }}_{diff}+\lambda_{1} & {k^{\prime }}_{diff}+\lambda_{1}\\ {k^{\prime }}_{diff} & {k^{\prime }}_{diff} \end{array}) \end{array}$$

The system becomes $\left(\begin{array}{c} \left[H_{2}O_{2}\right]\\ {\left[H_{2}O_{2}\right]}_{out} \end{array})=V(\begin{array}{cc} Ae^{\lambda_{1}t} & 0\\ 0 & Be^{\lambda_{2}t} \end{array}\right)$

The resolution shows a bi-exponential expression:

$$\begin{array}{c} \left[H_{2}O_{2}\right]=({k^{\prime }}_{diff}+\lambda_{1})Ae^{\lambda_{1}t}+({k^{\prime }}_{diff}+\lambda_{2})Be^{\lambda_{2}t}+\frac{k_{prod}}{k_{enz}} \end{array}$$ (1)

$$\begin{array}{c} {\left[H_{2}O_{2}\right]}_{out}=k_{diff}^{\prime }Ae^{\lambda_{1}t}+k_{diff}^{\prime }Be^{\lambda_{2}t}+\frac{k_{prod}}{k_{enz}} \end{array}$$ (2)

with

$$\begin{array}{c} A=\frac{\left({\left[H_{2}O_{2}\right]}_{0}-\frac{k_{prod}}{k_{enz}}){k^{\prime }}_{diff}-({\left[H_{2}O_{2}\right]}_{out0}-\frac{k_{prod}}{k_{enz}})({k^{\prime }}_{diff}+\lambda_{2}\right)}{(\lambda_{1}-\lambda_{2}){k^{\prime }}_{diff}} \end{array}$$ (3)

$$\begin{array}{c} B=\frac{\left({\left[H_{2}O_{2}\right]}_{0}-\frac{k_{prod}}{k_{enz}}){k^{\prime }}_{diff}-({\left[H_{2}O_{2}\right]}_{out0}-\frac{k_{prod}}{k_{enz}})({k^{\prime }}_{diff}+\lambda_{1}\right)}{(\lambda_{2}-\lambda_{1}){k^{\prime }}_{diff}} \end{array}$$ (4)

In this first approach, [*H*_2_ *O*_2_]_0_ = 0 and [*H*_2_ *O*_2_]_*out*0_ = 0: initial concentrations are taken to be zero.

From the very beginning, $\left[H_{2}O_{2}\right]\approx ({k^{\prime }}_{diff}+\lambda_{1})A+({k^{\prime }}_{diff}+\lambda_{2})Be^{\lambda_{2}t}+\frac{k_{prod}}{k_{enz}}$ as *e*^λ_1_*t*^ ≈ 1 because |λ_1_| ≈ 0.

Therefore $A\approx -\frac{k_{prod}}{k_{enz}{k^{\prime }}_{diff}}$ and $B=\frac{k_{prod}}{{\left(k_{enz}+k_{diff}\right)}^{2}}$

$$\begin{array}{c} k_{diff}^{\prime }+\lambda_{1}=k_{diff}^{\prime }-\frac{k_{enz}}{\left(k_{enz}+k_{diff}\right)}k_{diff}^{\prime }=\frac{k_{diff}}{\left(k_{enz}+k_{diff}\right)}k_{diff}^{\prime }; \end{array}$$

$$\begin{array}{c} k_{diff}^{\prime }+\lambda_{2}\approx -(k_{enz}+k_{diff}); \end{array}$$

In conclusion: $\left[H_{2}O_{2}\right]\approx \frac{k_{prod}}{k_{enz}+k_{diff}}(1-e^{-(k_{enz}+k_{diff})t})$

**Fig 1:**
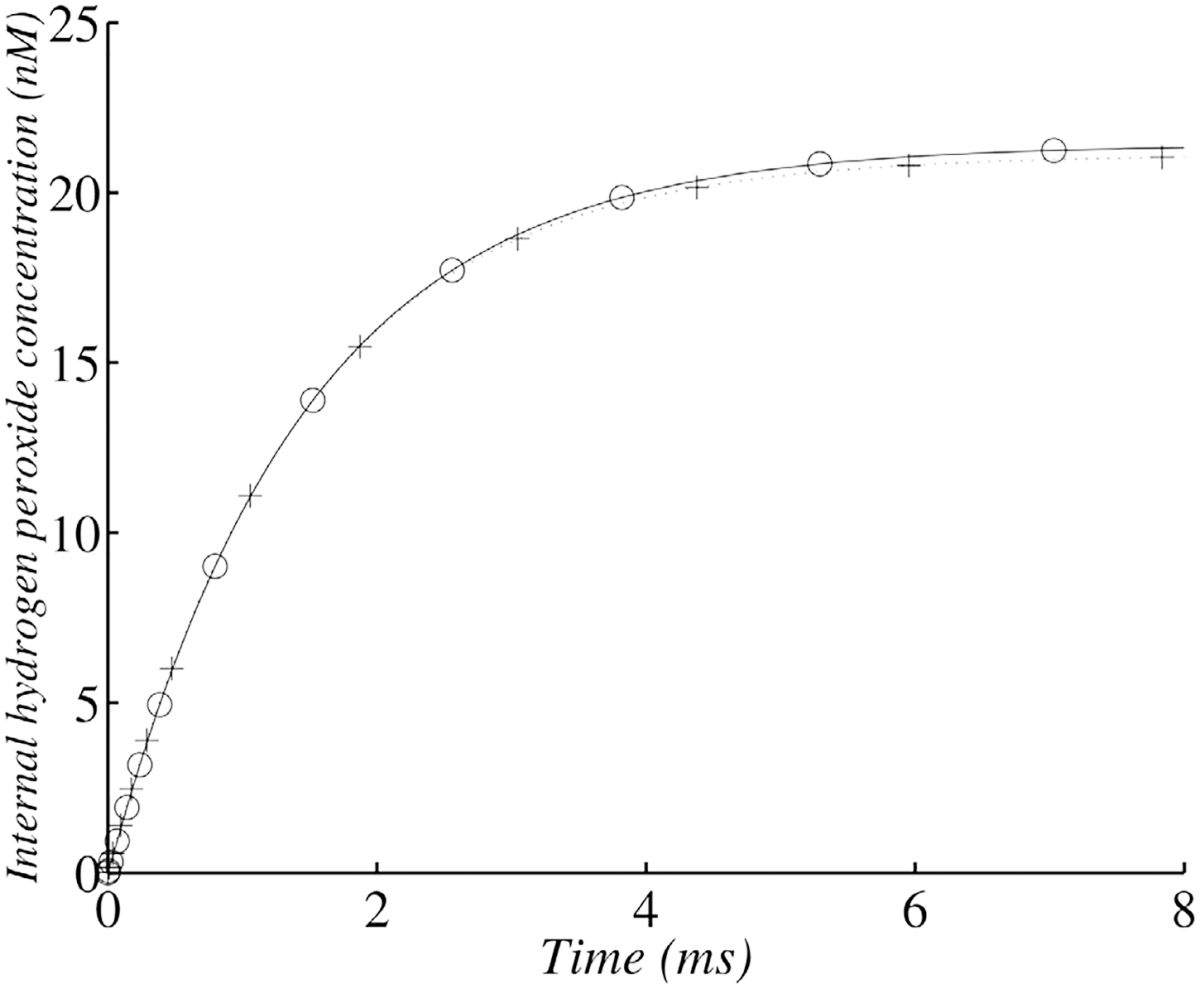
Changes in internal *H*_2_ *O*_2_ concentration in the wild-type strain with 10^7^ cells ml^−1^. (+) corresponds to the analytical solution of internal *H*_2_ *O*_2_ evolution according to the simplified system and (∘)) corresponds to the whole model solved with numerical methods.

The first plateau (in Fig 1) corresponds to the compromise between production and consumption, but consumption now also depends on diffusion across cell membrane. Indeed, the value of this first plateau is approximately $\frac{k_{prod}}{k_{enz}+k_{diff}}$.

The numerical values are ([4]):

$$\begin{array}{c} k_{enz}=633\;{\text{s}}^{-1}\;\text{with}\;\frac{k_{cat}^{Ahp}\left[Ahp\right]}{K_{M}^{Ahp}}=550\;{\text{s}}^{-1}\;;\frac{k_{cat}^{Kat}\left[Cat\right]}{K_{M}^{Kat}}=83\;{\text{s}}^{-1}\;;k_{diff}=70\;{\text{s}}^{-1} \end{array}$$

These values indicate that diffusion across the cell membrane accounts for approximately 10% of the *H*_2_ *O*_2_ eliminated, a level of activity close to that of Cat activity (≈12%). As previously reported ([5]), Ahp was identified as the principal scavenger (≈78%).

The first plateau concentration for *H*_2_ *O*_2_ is therefore $\frac{k_{prod}}{k_{enz}+k_{diff}}\approx 21$ nM.

For instance, in an Ahp(-) mutant without Cat induction, this concentration would be $\frac{k_{prod}}{\frac{k_{cat}^{Kat}\left[Cat\right]}{K_{M}^{Kat}}+k_{diff}}\approx 97$ nM.

After this transition step, we had *e*^λ_2_*t*^ ≈ 0. The change in *H*_2_ *O*_2_ concentration therefore follows this equation:

$$\begin{array}{c} \left[H_{2}O_{2}\right]=\frac{k_{prod}}{k_{enz}}-\frac{k_{prod}}{k_{enz}}\frac{k_{diff}}{\left(k_{enz}+k_{diff}\right)}e^{\lambda_{1}t} \end{array}$$

**Fig 2:**
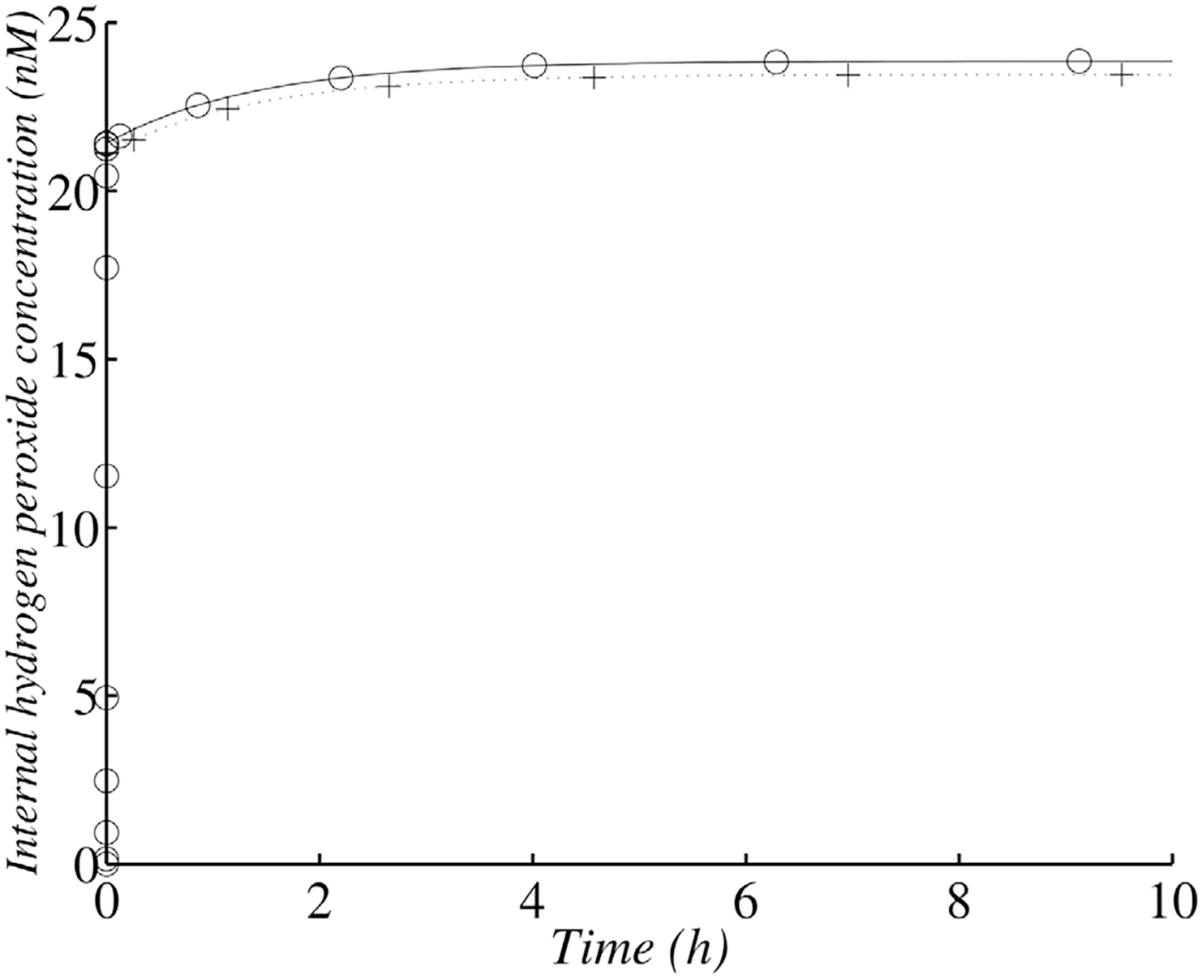
Changes in internal *H*_2_ *O*_2_ concentration in the wild-type strain with 10^6^ cells ml^−1^. (+) corresponds to the analytical solution according to the simplified system and (∘)) corresponds to the whole model and a numerical solution.

**Fig 3:**
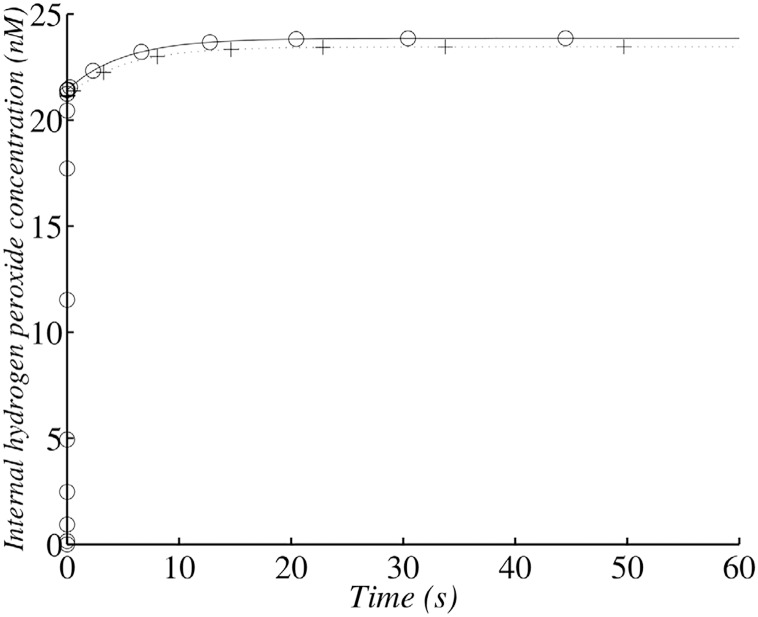
Changes in *H*_2_ *O*_2_ concentration in the wild-type strain with 10^9^ cells ml^−1^. (+) corresponds to the analytical solution according to the simplified system and (∘)) corresponds to the whole model and a numerical solution.

This second step is slower and depends on the number of cells, with the final steady-state concentration of *H*_2_ *O*_2_ reached more rapidly for denser cell populations (Figs 2 and 3).

The final steady-state value is ${\left[H_{2}O_{2}\right]}_{\infty }=\frac{k_{prod}}{k_{enz}}\approx 23.5$ nM and is not dependent on cell number. This value is close to that obtained by numerical simulation (23.9 nM) and to that proposed by Imlay (20 nM) ([4]).

For instance, in an Ahp(-) mutant without Cat induction, this value would be $\frac{k_{prod}K_{M}^{Kat}}{k_{cat}^{Kat}\left[Cat\right]}\approx 179$ nM (identical to the numerical simulation value and close to the value of 100 nM proposed by Seaver and Imlay ([4]).

This second step in the change in *H*_2_ *O*_2_ concentration depends on $\lambda_{1}\approx -\frac{k_{enz}}{\left(k_{enz}+k_{diff}\right)}k_{diff}^{\prime }$, which depends on cell concentration *via* the $k_{diff}^{\prime }$.

The results are summarized in Table 2.

**Table 2:** *H*_2_ *O*_2_ steady-state concentration.

[*H*_2_ *O*_2_] (nmol L^−1^)	In this work	Seaver, Imlay [4]
Wild type	24	21
HPI+ HPII+ Ahp- (*AhpCF*^(*a*)^)	179	100
HPI- HPII- Ahp+ (*KatEKatG*)	28	23

At steady state, the internal concentration is shown for cells in LB at 37°C.

(a) without induced HPI levels.

#### With exogenous stress

We propose linear approximations of Michaelis-Menten kinetics. Internal *H*_2_ *O*_2_ concentration approximately follows the law outlined below. Let us consider an experiment involving the addition of exogenous *H*_2_ *O*_2_. The initial *ROS* concentrations in the cell are taken to be the steady-state values obtained without exogenous *H*_2_ *O*_2_. The system requires modification as follows:

d[H2O2]dt=k1′+12k2[SOD][O2•-]-kcatAhp[Ahp][H2O2][H2O2]+KMAhp-kcatKat[Cat][H2O2][H2O2]+KMKat-kdiff([H2O2]-[H2O2]out)d[H2O2]outdt=kdiffn·VinVout-nVin([H2O2]-[H2O2]out)

As the system is nonlinear there is no analytical solution so with a view to solving the system, we had to compare the value obtained for the internal concentration of *H*_2_ *O*_2_ with the *K_*M*_* values of Ahp and Cat to simplify the Michaelis-Menten expression. Moreover, cell behavior (and thus the dynamic system) depends on the comparison of internal *H*_2_ *O*_2_ concentration with the *K_*M*_* values of Ahp and Cat. This comparison is essential to simplify the system into a linear one, which will then be solvable. This kind of study is frequently carried out and provides useful insight into the workings of systems. For example, Polynikis *et* *al*. ([18]) compared different modeling approaches (complete nonlinear model, simplified piecewise linear model etc.) for gene regulatory networks using Hill functions, a general form of the Michaelis-Menten equation.

To approximate the Michaelis-Menten hyperbole into a piecewise linear function, let us first examine the contribution of the two enzymes.

**Fig 4:**
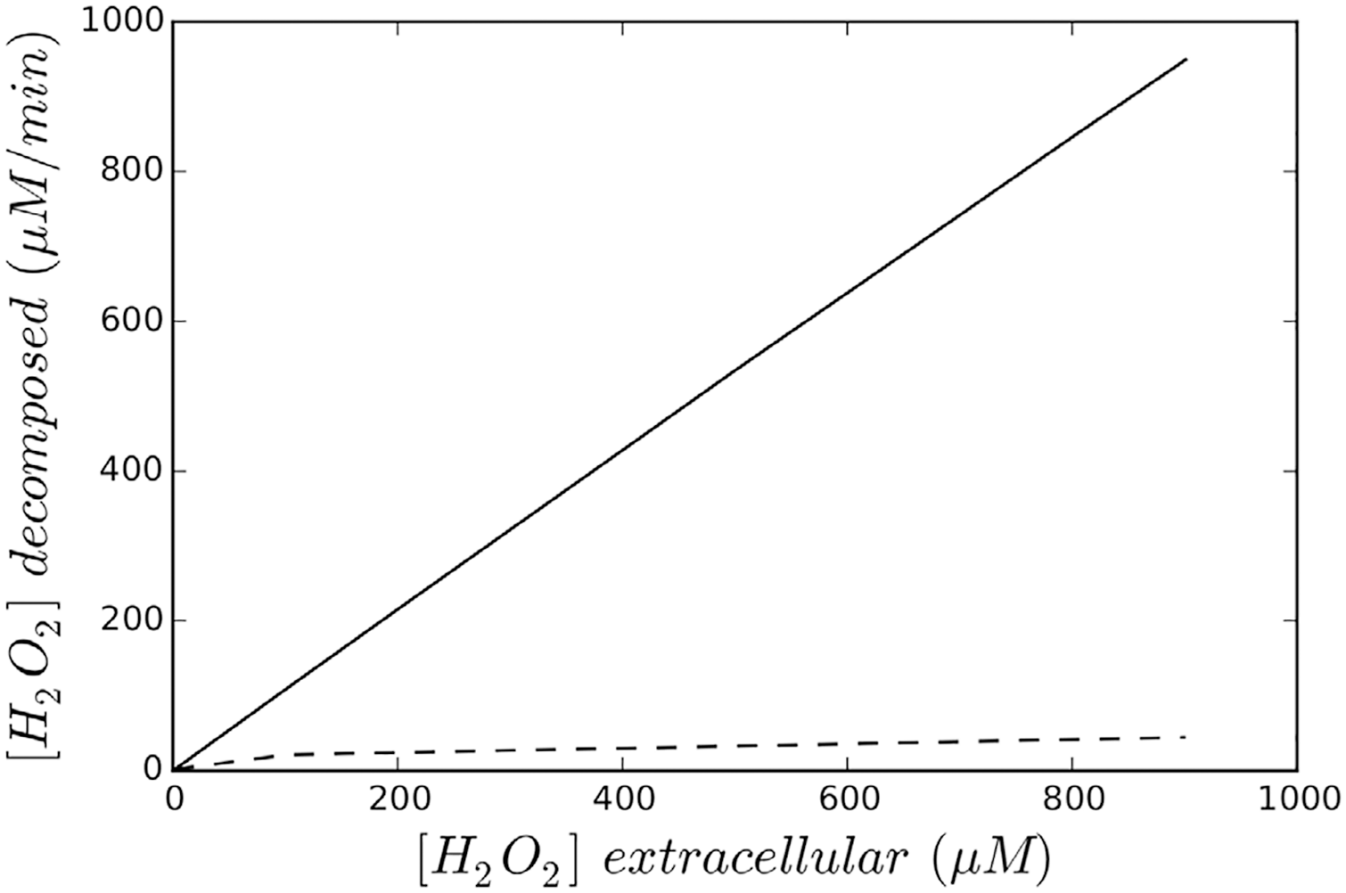
Kinetics of exogenous *H*_2_ *O*_2_ decomposition for 1.5 × 10^8^ *E. coli* cells ml^−1^. The dotted line corresponds to the Cat(-) mutant and solid line corresponds to the Ahp (-) mutant.

**Fig 5:**
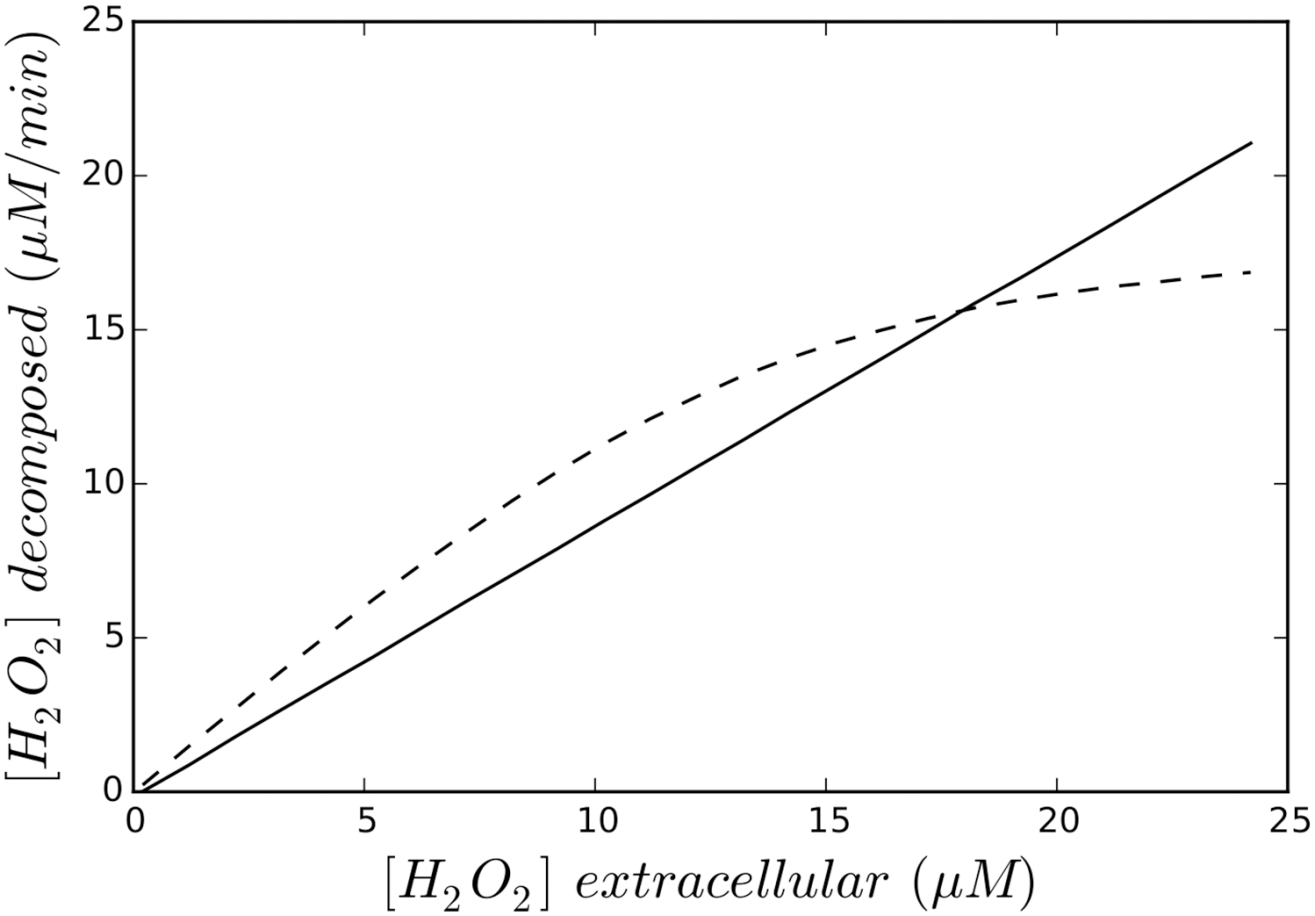
Kinetics of exogenous *H*_2_ *O*_2_ decomposition for 1.5 × 10^8^ *E. coli* cells ml^−1^. The dotted line corresponds to the Cat(-) mutant and solid line corresponds to the Ahp (-) mutant (higher magnification for Fig 4).

The rate (see Fig 4) followed the same pattern of change as that presented by Seaver and Imlay ([5]). Ahp was the leading scavenger in conditions of 17 *μ*M exogenous *H*_2_ *O*_2_ (see intersection point in Fig 5).

We can consider that, in the presence of less than 10 *μ*M *H*_2_ *O*_2_, Ahp activity is linear (Fig 5) and that Cat activity is linear at concentrations below 10 mM (due to its K_*M*_ value). At *H*_2_ *O*_2_ concentrations of more than 30 *μ*M, Ahp activity is saturated.

According to the Michaelis-Menten equation, we should consider Ahp activity to be linear when $\left[H_{2}O_{2}\right]⪡K_{M}^{Ahp}\approx 1$ *μ*M, but linearity was observed when [*H*_2_ *O*_2_]_*out*_ < 10 *μ*M. It is unclear why there is a difference of one order of magnitude between exogenous [*H*_2_ *O*_2_]_*out*_ and internal [*H*_2_ *O*_2_] at the limit of linearity.

Such a difference was reported in another experiment presented by Seaver and Imlay ([4]) while studying *H*_2_ *O*_2_ fluxes.

**Fig 6:**
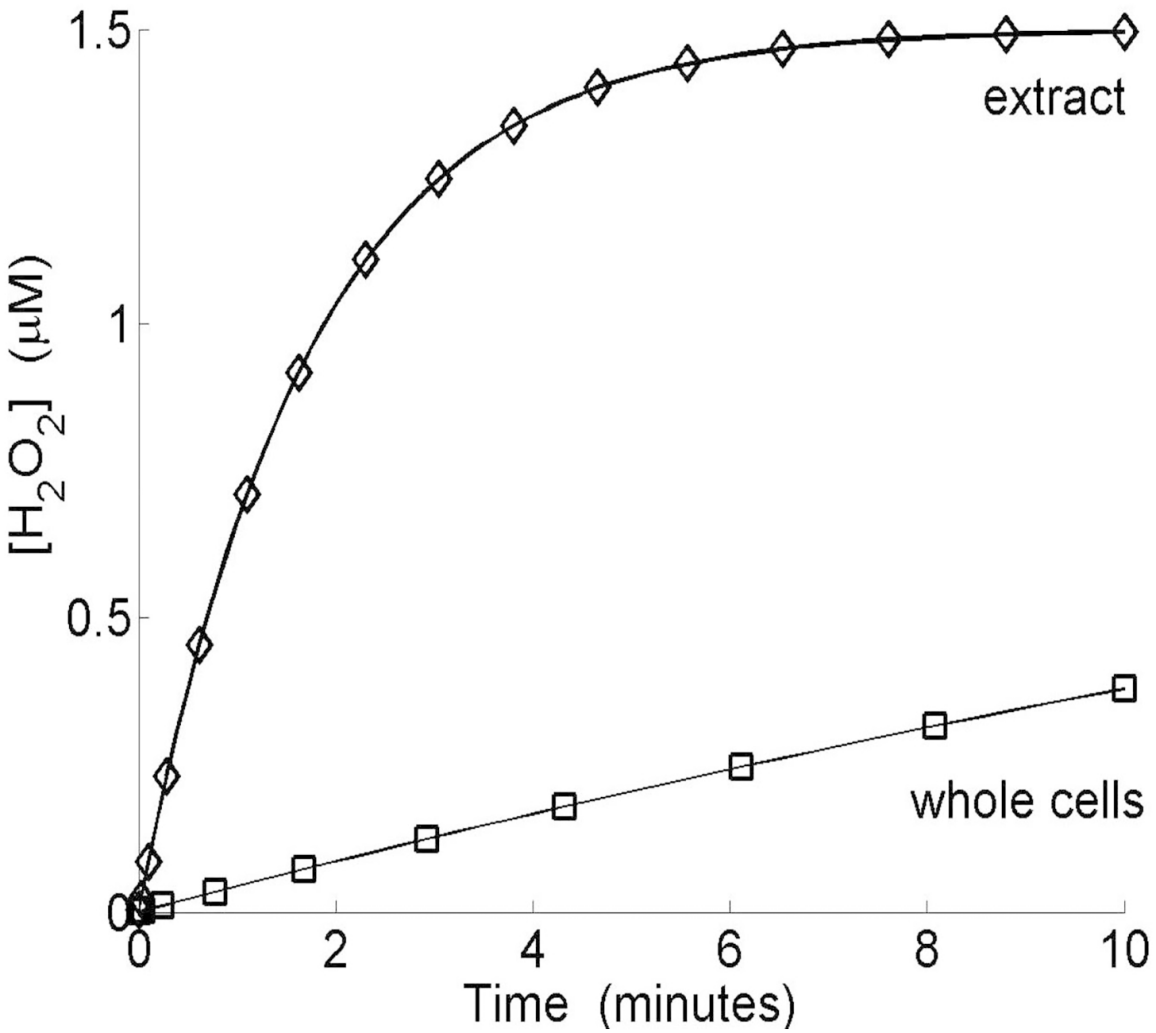
*In silico*, breakdown of exogenous *H*_2_ *O*_2_ by whole cells (Ahp(-) mutant) or cell extract. Simulation runs with 4 × 10^6^ cells ml^−1^. Moreover, Seaver and Imlay ([5]) observed that an Ahp(-) mutant contained seven times as much total Cat as wild-type cells. We therefore used the same ratio.

In this experiment, whole cells seemed to scavenge *H*_2_ *O*_2_ less efficiently than cell extracts. The cell membrane slows the entry of *H*_2_ *O*_2_, resulting in lower rates of decomposition. It also protects cells against high *H*_2_ *O*_2_ concentrations, by decreasing the maximum value of *H*_2_ *O*_2_ concentration. This phenomenon is described in more detail below. The simulation (Fig 6) for extract was modeled by eliminating membrane diffusion and the metabolism associated with *ROS* production. There is a perfect match between numerical simulation and the experimental results of Seaver and Imlay.

Two situations can be distinguished based on these previous observations.

In the first case, $\left[H_{2}O_{2}\right]⪡K_{M}^{Ahp}$ corresponds to [*H*_2_ *O*_2_]_*out*_ < 10 *μ*M and to $\left[H_{2}O_{2}\right]⪡K_{M}^{Cat}$. The system approaches Michaelis-Menten terms as follows:

$$\begin{array}{ccc} \frac{k_{cat}^{Ahp}\left[Ahp\right]\left[H_{2}O_{2}\right]}{\left[H_{2}O_{2}\right]+K_{M}^{Ahp}}+\frac{k_{cat}^{Kat}\left[Cat\right]\left[H_{2}O_{2}\right]}{\left[H_{2}O_{2}\right]+K_{M}^{Kat}} & \approx & \frac{k_{cat}^{Ahp}\left[Ahp\right]}{K_{M}^{Ahp}}+\frac{k_{cat}^{Kat}\left[Cat\right]}{K_{M}^{Kat}}\left[H_{2}O_{2}\right]\\ & = & k_{enz}\left[H_{2}O_{2}\right] \end{array}$$

In the second case, if $\left[H_{2}O_{2}\right]\gg K_{M}^{Ahp}$, corresponding to [*H*_2_ *O*_2_]_*out*_ > 30 *μ*M and to $\left[H_{2}O_{2}\right]⪡K_{M}^{Cat}$, then the system approaches Michaelis-Menten terms as follows:

$$\begin{array}{ccc} \frac{k_{cat}^{Ahp}[Ahp][H_{2}O_{2}]}{[H_{2}O_{2}]+K_{M}^{Ahp}}+\frac{k_{cat}^{Kat}[Cat][H_{2}O_{2}]}{[H_{2}O_{2}]+K_{M}^{Kat}} & \approx & \left(k_{cat}^{Ahp}[Ahp]+\frac{k_{cat}^{Kat}[Cat]}{K_{M}^{Kat}})[H_{2}O_{2}\right]\\ & = & k_{cat}^{Ahp}[Ahp]+k_{enz}^{\prime }[H_{2}O_{2}] \end{array}$$

where $\frac{k_{cat}^{Kat}\left[Cat\right]}{K_{M}^{Kat}}=k_{enz}^{\prime }$.

Then we examine Ahp activity with a micromolar exogenous *H*_2_ *O*_2_ concentration. In the first case ($\left[H_{2}O_{2}\right]⪡K_{M}^{Ahp}$ and $\left[H_{2}O_{2}\right]⪡K_{M}^{Cat}$), the differential equation system appears to be the same as that without exogenous *H*_2_ *O*_2_, but [*H*_2_ *O*_2_]_*out*_ ≠ 0. As ${\left[H_{2}O_{2}\right]}_{0}=\frac{k_{prod}}{k_{enz}}$, the constants *A* and *B* can be simplified as follows:

$$\begin{array}{c} A=\frac{-\left({\left[H_{2}O_{2}\right]}_{out0}-\frac{k_{prod}}{k_{enz}}\right)\left({k^{\prime }}_{diff}+\lambda_{2}\right)}{(\lambda_{1}-\lambda_{2}){k^{\prime }}_{diff}} \end{array}$$

and

$$\begin{array}{c} B=\frac{-({\left[H_{2}O_{2}\right]}_{out0}-\frac{k_{prod}}{k_{enz}})({k^{\prime }}_{diff}+\lambda_{1})}{(\lambda_{2}-\lambda_{1}){k^{\prime }}_{diff}} \end{array}$$

Moreover as $\lambda_{1}\approx -\frac{k_{enz}}{\left(k_{enz}+k_{diff}\right)}k_{diff}^{\prime }$ and λ_2_ ≈ −(*k*_*enz*_ + *k*_*diff*_) with |λ_1_| < <|λ_2_| and |*k*′_*diff*_| < <|λ_2_|

$$\begin{array}{c} \left({k^{\prime }}_{diff}+\lambda_{1})A\approx -({k^{\prime }}_{diff}+\lambda_{2}\right) \end{array}$$

$$\begin{array}{c} B\approx ({\left[H_{2}O_{2}\right]}_{out0}-\frac{k_{prod}}{k_{enz}})\frac{k_{diff}}{k_{diff}+k_{enz}} \end{array}$$

therefore:

$$\begin{array}{c} {}[H_{2}O_{2}]=({\left[H_{2}O_{2}\right]}_{out0}-\frac{k_{prod}}{k_{enz}})\frac{k_{diff}}{k_{diff}+k_{enz}}(e^{\lambda_{1}t}-e^{\lambda_{2}t})+\frac{k_{prod}}{k_{enz}} \end{array}$$

This bi-exponential function shows that changes in internal *H*_2_ *O*_2_ concentration follow two phases. There is a first phase, with a large rate constant −λ_2_ ≈ *k*_*enz*_ + *k*_*diff*_ corresponding to the scavenging process, followed by a much slower second phase, with a low rate constant −λ_1_ > 0 corresponding to the diffusion from the external *H*_2_ *O*_2_ into the cell. This second phase is faster for larger numbers of cells because $k_{diff}^{\prime }$ is highly dependent on cell concentration.

$-\lambda_{1}\approx \frac{k_{enz}}{\left(k_{enz}+k_{diff}\right)}k_{diff}^{\prime }$ and as *k*_*enz*_ ≫ *k*_*diff*_ we can approach $-\lambda_{1}\approx k_{diff}^{\prime }$.

This function therefore reaches a maximum as $\frac{d\left[H_{2}O_{2}\right]}{dt}=0$ for:

$$\begin{array}{c} t_{\max}=\frac{1}{\lambda_{2}-\lambda_{1}}\ln(\frac{\lambda_{1}}{\lambda_{2}})\approx \frac{1}{\left(k_{enz}+k_{diff}\right)}\ln(\frac{{\left(k_{enz}+k_{diff}\right)}^{2}}{k_{enz}{k^{\prime }}_{diff}}) \end{array}$$

This time is weakly dependent on cell numbers. For example, *t*_max_ ≈ 18 ms with 10^7^ cells ml^−1^ and 11 ms with 10^9^ cells ml^−1^.

The maximum internal *H*_2_ *O*_2_ concentration is approximately:

$$\begin{array}{c} {\left[H_{2}O_{2}\right]}_{\max}\approx ({\left[H_{2}O_{2}\right]}_{out0}-\frac{k_{prod}}{k_{enz}})\frac{k_{diff}}{k_{diff}+k_{enz}}+\frac{k_{prod}}{k_{enz}} \end{array}$$

and as ${\left[H_{2}O_{2}\right]}_{out0}\gg \frac{k_{prod}}{k_{enz}}={\left[H_{2}O_{2}\right]}_{\infty }$ which can be approached by:

$$\begin{array}{c} {\left[H_{2}O_{2}\right]}_{\max}\approx \frac{k_{diff}{\left[H_{2}O_{2}\right]}_{out0}}{k_{diff}+k_{enz}} \end{array}$$

therefore

$$\begin{array}{c} \frac{{\left[H_{2}O_{2}\right]}_{\max}}{{\left[H_{2}O_{2}\right]}_{out0}}\approx \frac{1}{1+k_{enz}/k_{diff}}\approx \frac{1}{9} \end{array}$$

The balance between the elimination processes in the value of the maximal internal *H*_2_ *O*_2_ concentration is due to:

$$\begin{array}{c} \frac{k_{cat}^{Ahp}[Ahp]}{K_{M}^{Ahp}(k_{enz}+k_{diff})}\approx 78\%\;\text{to}\;\text{Ahp} \end{array}$$

$$\begin{array}{c} \frac{k_{cat}^{Kat}[Cat]}{K_{M}^{Kat}(k_{enz}+k_{diff})}\approx 12\%\;\text{to}\;\text{Cat} \end{array}$$

and

$$\begin{array}{c} \frac{k_{diff}}{k_{enz}+k_{diff}}\approx 10\%\\ \text{to elimination by diffusion throughout cell membrane} \end{array}$$

The maximal value of internal *H*_2_ *O*_2_ concentration is almost one tenth the initial exogenous *H*_2_ *O*_2_ concentration. This phenomenon reflects the role of the cell membrane in limiting diffusion. The need to diffuse across the cell membrane limits the influx of exogenous *H*_2_ *O*_2_ and this process is highly effective at low exogenous *H*_2_ *O*_2_ concentrations. The difference of one order of magnitude between exogenous [*H*_2_ *O*_2_]_*out*_ and internal [*H*_2_ *O*_2_] arises because the membrane creates a rate-limiting step.

To illustrate this, we will investigate cell behaviour in the presence of 1.5 *μ*M exogenous *H*_2_ *O*_2_.

**Fig 7:**
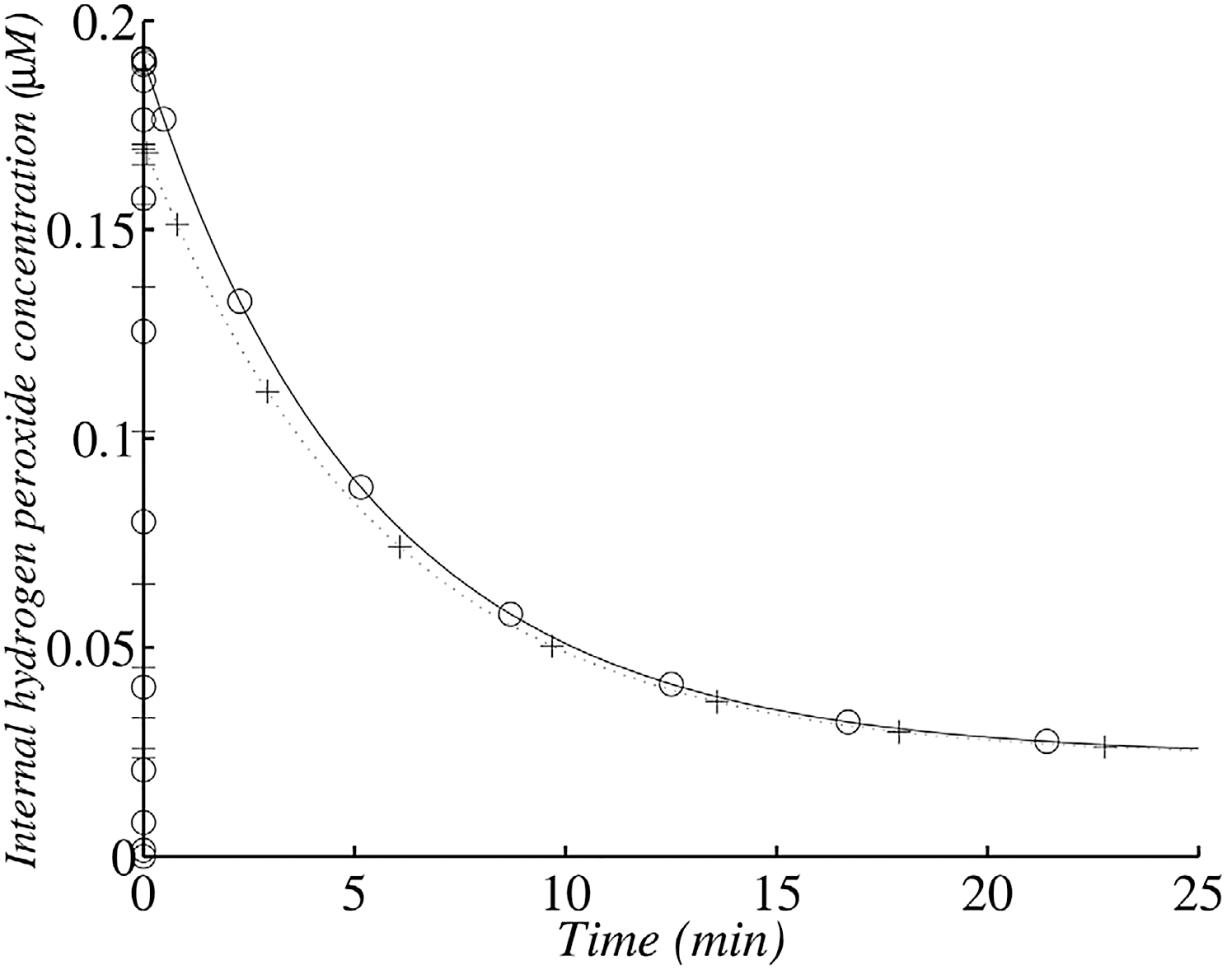
Changes in internal *H*_2_ *O*_2_ concentration in the wild type after the addition of 1.5 *μ*M exogenous *H*_2_ *O*_2_ with 1.45 × 10^7^ cells ml^−1^. (+) corresponds to the analytical solution according to the simplified system and (∘) corresponds to the numerical solution of the whole model. The simulation was run with 1.45 × 10^7^ cells ml^−1^ (corresponding to an OD_600_ value of 0.1).

After its peak value (Fig 7), internal *H*_2_ *O*_2_ concentration decreases because of scavenging, but diffusion across cell membrane is the process which limits the rate of *H*_2_ *O*_2_ disappearance, therefore *H*_2_ *O*_2_ decrease is slow. The membrane creates a rate-limiting step.

The maximal value is the approximate value of the first plateau proposed by Gonzalez-Flecha and Demple ([19]). It corresponds to the ratio of the rate of *H*_2_ *O*_2_ influx by diffusion to levels of scavenging and elimination by diffusion.

The experiments of Seaver and Imlay ([5]) showed that even non-induced cells scavenged micromolar concentrations of exogenous *H*_2_ *O*_2_ very quickly. For example, in a culture corresponding to 0.1 OD_680_ unit (corresponding to around 1.5 × 10^7^ cells ml^−1^), they found that the half time of *H*_2_ *O*_2_ in the medium was only 3.5 minutes, and that in a culture of 1.0 OD unit it was 20 s.

The exogenous *H*_2_ *O*_2_ concentration approximately follows the law outlined below:

$$\begin{array}{c} {\left[H_{2}O_{2}\right]}_{out}=({\left[H_{2}O_{2}\right]}_{0}-\frac{k_{prod}}{k_{enz}})e^{\lambda_{1}t}+\frac{k_{prod}}{k_{enz}} \end{array}$$

where

$$\begin{array}{c} \lambda_{1}\approx -\frac{k_{enz}}{\left(k_{enz}+k_{diff}\right)}k_{diff}^{\prime }\approx k_{diff}^{\prime } \end{array}$$

**Fig 8:**
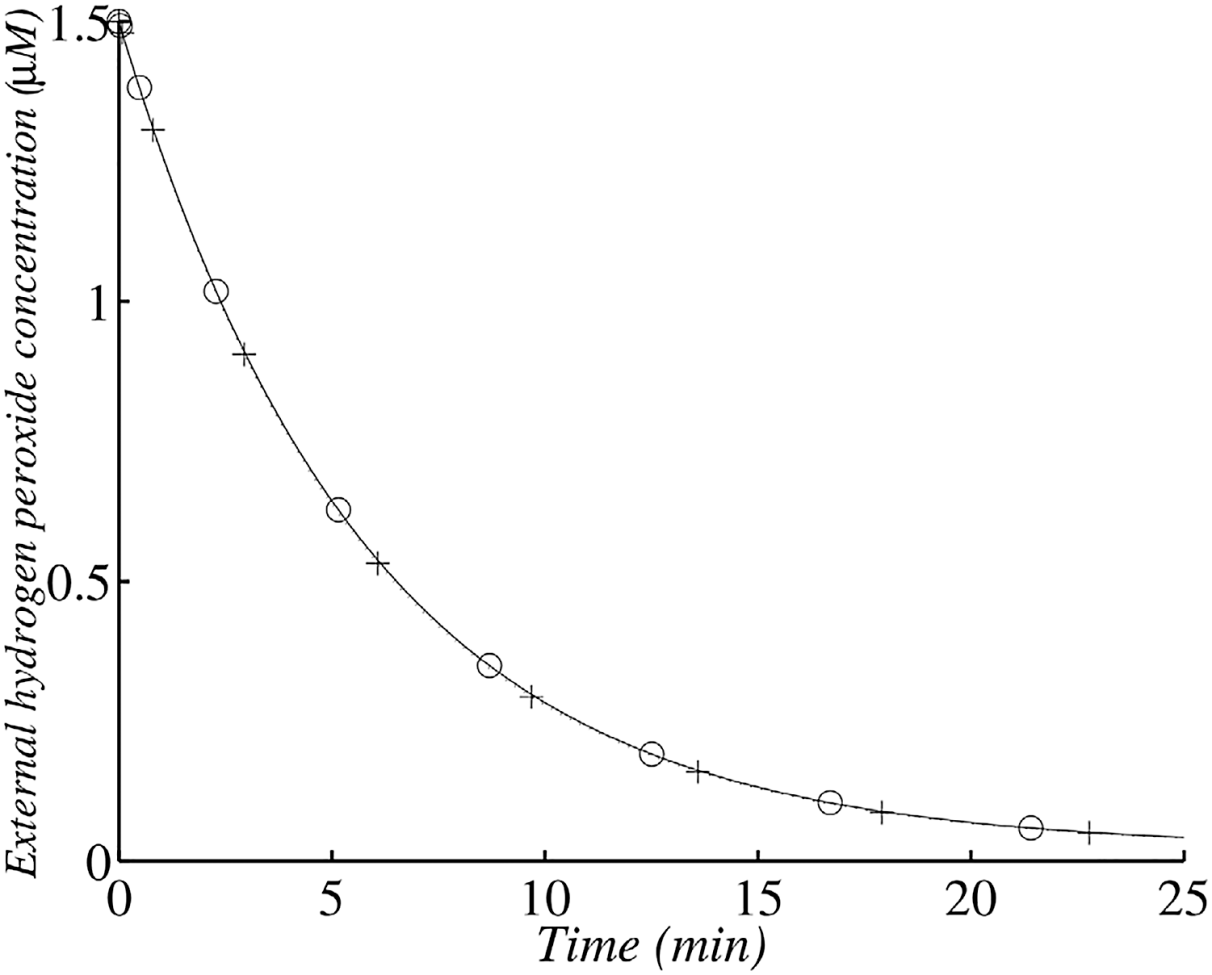
Changes in external *H*_2_ *O*_2_ concentration in the wild type after the addition of 1.5 *μ*M exogenous *H*_2_ *O*_2_ with 1.45 × 10^7^ cells ml^−1^. (+) corresponds to the analytical solution according to the simplified system and (∘)) corresponds to the numerical solution of the whole model. The simulation was run with 1.45 × 10^7^ cells ml^−1^ (corresponding to an OD_680_ value of 0.1).

**Fig 9:**
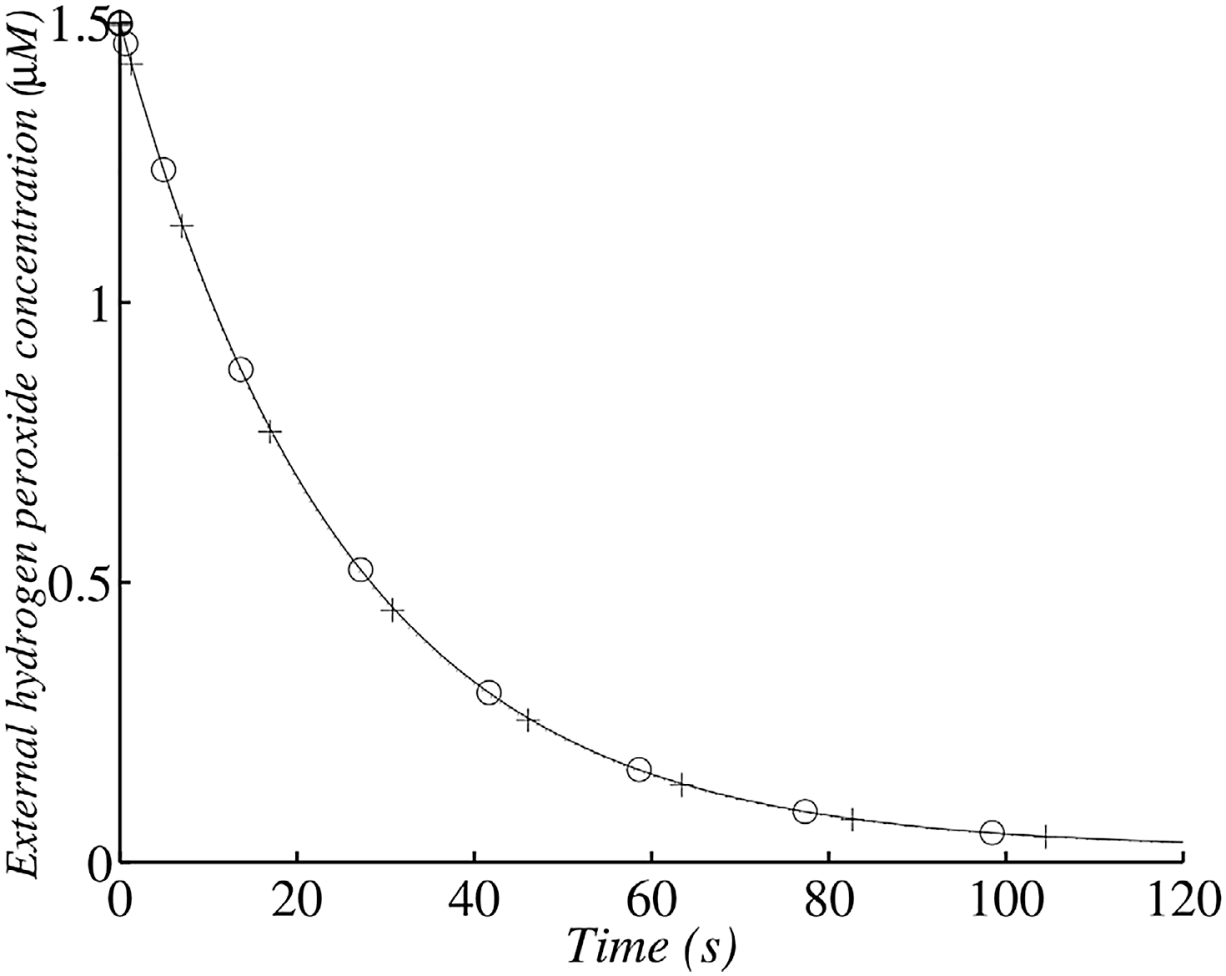
Changes in external *H*_2_ *O*_2_ concentration in the wild type after the addition of 1.5 *μ*M exogenous *H*_2_ *O*_2_ with 2 × 10^8^ cells ml^−1^. (+) corresponds to the analytical solution according to the simplified system and (∘)) corresponds to the numerical solution of the whole model. The simulation was run with 2 × 10^8^ cells ml^−1^ (corresponding to an OD_680_ value of 0.1).

Its exponential decrease depends on the $k_{diff}^{\prime }$ rate constant, which is strongly dependent on cell numbers. The half-life of *H*_2_ *O*_2_ in the medium is approximately $t_{1/2}\approx \frac{\ln2}{k_{diff}^{\prime }}$ and is a decreasing function of cell number. So, with an OD_680_ of 0.1 (Fig 8) we find that *t*_1/2_ ≈ 210 s (3.5 min) and with an OD_680_ of 1 we find that *t*_1/2_ ≈ 21 s (Fig 9). A comparison of the experimental data and the analytical results indicates that our model describes the change in *H*_2_ *O*_2_ correctly.

Finally we examine Ahp activitys, with a high *H*_2_ *O*_2_ concentration. In the second case, when $\left[H_{2}O_{2}\right]\gg K_{M}^{Ahp}$, corresponding to [*H*_2_ *O*_2_]_*out*_ > 30 *μ*M, the differential equation system can be written with a matrix structure:

$$\begin{array}{c} \frac{d}{dt}(\begin{array}{c} \left[H_{2}O_{2}\right]\\ {\left[H_{2}O_{2}\right]}_{out} \end{array})=(\begin{array}{c} {k^{\prime }}_{prod}\\ 0 \end{array})+(\begin{array}{cc} -({k^{\prime }}_{enz}+k_{diff}) & k_{diff}\\ {k^{\prime }}_{diff} & -{k^{\prime }}_{diff} \end{array})(\begin{array}{c} \left[H_{2}O_{2}\right]\\ {\left[H_{2}O_{2}\right]}_{out} \end{array}) \end{array}$$

where kprod′=k1′+12k1-kcatAhp[Ahp] is the usual production reduced by Ahp activity on saturation; and $k_{enz}^{\prime }=\frac{k_{cat}^{Kat}\left[Cat\right]}{K_{M}^{Kat}}$ (only Cat follows linear kinetics)

The study is similar to the previous one and internal *H*_2_ *O*_2_ concentration can be expressed as follows:

$$\begin{array}{c} {}[H_{2}O_{2}]=({\left[H_{2}O_{2}\right]}_{out0}-\frac{{k^{\prime }}_{prod}}{{k^{\prime }}_{enz}})\frac{k_{diff}}{k_{diff}+{k^{\prime }}_{enz}}(e^{{\lambda^{\prime }}_{1}t}-e^{{\lambda^{\prime }}_{2}t})+\frac{{k^{\prime }}_{prod}}{{k^{\prime }}_{enz}} \end{array}$$

with the eigenvalue $\lambda_{1}\approx -\frac{{k^{\prime }}_{enz}}{\left({k^{\prime }}_{enz}+k_{diff}\right)}k_{diff}^{\prime }$ and λ_2_ ≈ −(*k*′_*enz*_ + *k*_*diff*_)

The maximum will be ${\left[H_{2}O_{2}\right]}_{\max}\approx \frac{k_{diff}{\left[H_{2}O_{2}\right]}_{out0}}{k_{diff}+{k^{\prime }}_{enz}}$. With large concentrations of exogenous *H*_2_ *O*_2_, ${\left[H_{2}O_{2}\right]}_{\max}\approx \frac{k_{diff}{\left[H_{2}O_{2}\right]}_{out0}}{k_{diff}+\frac{k_{cat}^{Kat}\left[Cat\right]}{K_{M}^{Kat}}}$. This corresponds to the ratio of the rate of *H*_2_ *O*_2_ influx by diffusion to its elimination by Cat or by diffusion only.

This expression shows that the ratio between the initial exogenous *H*_2_ *O*_2_ concentration and the maximal internal *H*_2_ *O*_2_ concentration in the cell is:

$$\begin{array}{c} \frac{{\left[H_{2}O_{2}\right]}_{\max}}{{\left[H_{2}O_{2}\right]}_{out0}}\approx \frac{1}{1+\frac{k_{cat}^{Kat}[Cat]}{k_{diff}K_{M}^{Kat}}}\approx \frac{1}{2.2}\left(*\right) \end{array}$$

The contribution of each elimination process to the value of the maximal internal *H*_2_ *O*_2_ concentration is:

$$\begin{array}{c} \frac{{k^{\prime }}_{enz}}{{k^{\prime }}_{enz}+k_{diff}}\approx 55\%\text{to Cat}\\ \text{and}\;\frac{k_{diff}}{{k^{\prime }}_{enz}+k_{diff}}\approx 45\;\%\;\text{to elimination by diffusion across the cell membrane} \end{array}$$

We notice that: $\frac{{\left[H_{2}O_{2}\right]}_{\max}}{{\left[H_{2}O_{2}\right]}_{out0}}$ is equal to the ratio of elimination by diffusion across the membrane to the sum diffusion and scavenging. Of course, without membrane this ratio will equal 1, so thanks to membrane, enzymes have to face less *H*_2_ *O*_2_. Moreover, at high exogenous *H*_2_ *O*_2_ concentrations, this ratio is quite different from the one (i.e. 1/9) obtained at low concentration.

**Fig 10:**
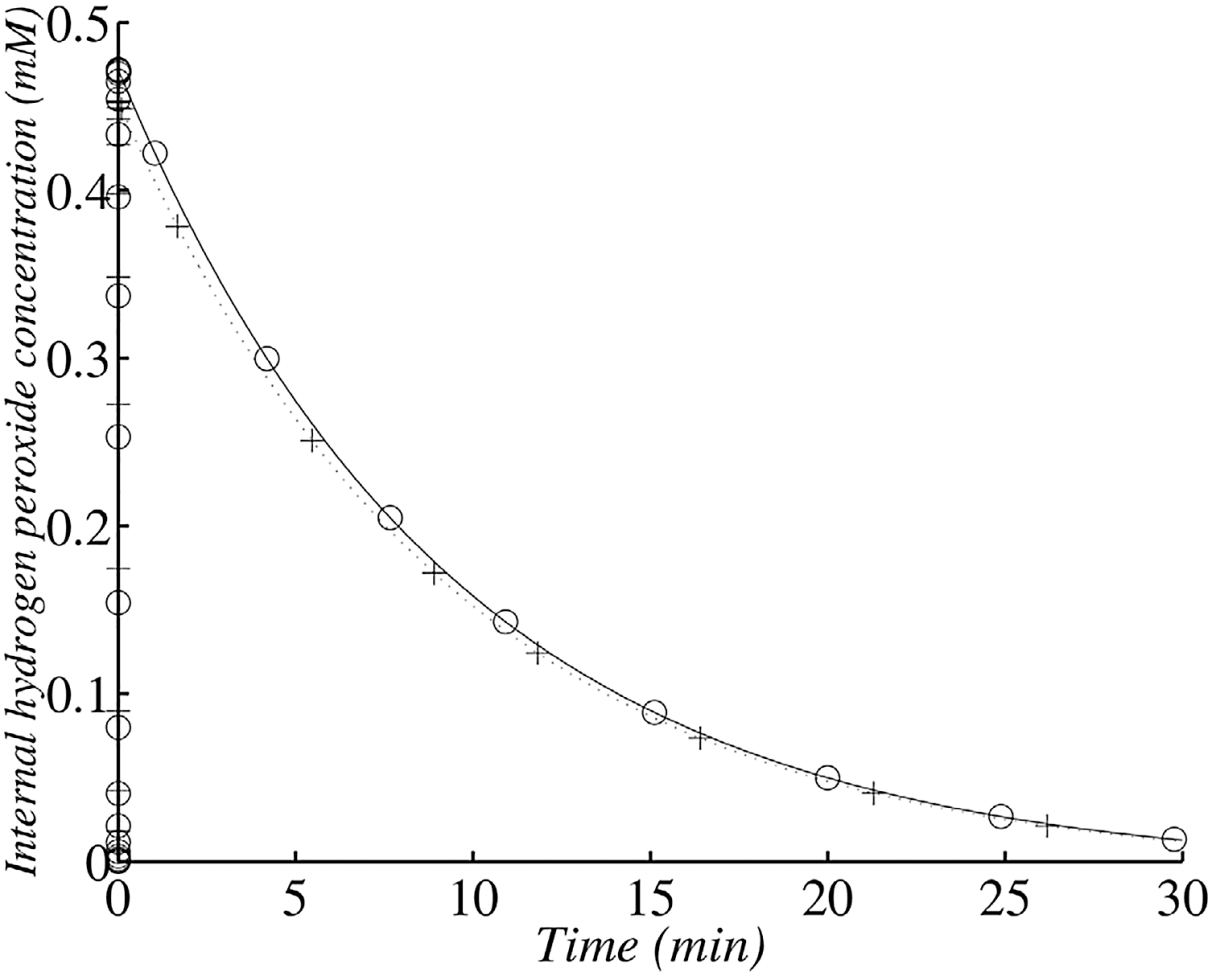
Changes in internal *H*_2_ *O*_2_ concentration in the wild type after the addition of 1 mM exogenous *H*_2_ *O*_2_ with 1.45 × 10^7^ cells ml^−1^. (+) corresponds to the analytical solution according to the simplified system and (∘)) corresponds to the numerical solution of the whole model.

For instance, with an initial *H*_2_ *O*_2_ exogenous concentration [*H*_2_ *O*_2_]_*out*0_ = 1 mM, we obtain [*H*_2_ *O*_2_]_max_ ≈ 0.45 mM (Fig 10). The maximal value is lower than the exogenous concentration because of diffusion and Cat activity, in this case Ahp is saturated and therefore plays a less important role.

The exogenous *H*_2_ *O*_2_ concentration approximately follows the law outlined below:

$$\begin{array}{c} {\left[H_{2}O_{2}\right]}_{out}=({\left[H_{2}O_{2}\right]}_{0}-\frac{k_{prod}}{k_{enz}})e^{{\lambda^{\prime }}_{1}t}+\frac{k_{prod}}{k_{enz}} \end{array}$$

where

$$\begin{array}{c} \lambda_{1}^{\prime }\approx -\frac{{k^{\prime }}_{enz}}{\left({k^{\prime }}_{enz}+k_{diff}\right)}k_{diff}^{\prime }\neq k_{diff}^{\prime } \end{array}$$

This exponential decrease depends on $\lambda_{1}^{\prime }$, which is a cell density function. The decrease rate of *H*_2_ *O*_2_ can be characterized by the half-time *t*_1/2_. This time is approximately the same for internal and external concentration, as internal and external *H*_2_ *O*_2_ decrease are strongly linked. For instance, with an addition of 1 mM of exogenous *H*_2_ *O*_2_ and with a cell density of 1.45 × 10^7^ cells ml^−1^, the half-live is approximately $t_{1/2}\approx \frac{\ln1/2}{\lambda_{1}^{\prime }}\approx 6.5$ minutes, this results is consistent with Fig 10.

Moreover, as the exponential decrease in rate is dependent on $\lambda_{1}^{\prime }$, it ranges from zero when there is no scavenger (in a cat- mutant) to $k_{diff}^{\prime }$ when scavengers have a non-limiting rate constant (much higher than *k*_*diff*_). Thus, a 10-fold induction of Cat (experimentally observed in an Ahp(-) mutant) should increase the rate of medium detoxification of high *H*_2_ *O*_2_ concentrations only with a ratio of:

$$\begin{array}{c} \frac{{\lambda^{\prime }}_{1,induction}}{{\lambda^{\prime }}_{1}}=\lambda_{1}^{\prime }\approx \frac{10({k^{\prime }}_{enz}+k_{diff})}{\left(10{k^{\prime }}_{enz}+k_{diff}\right)}\approx 1.7 \end{array}$$

This result is consistent with the experimental data of Seaver and Imlay ([5]), who examined a doubling in efficiency when comparing the wild type and an Ahp(-) mutant.

It should also be noted that, in a Cat(-) mutant [*H*_2_ *O*_2_]_max_ ≈ [*H*_2_ *O*_2_]_*out*0_ ≈ [*H*_2_ *O*_2_]_0_ (according to equation *). The maximum internal *H*_2_ *O*_2_ concentration rapidly increases the exogenous *H*_2_ *O*_2_ concentration and, as there is no Cat, this value remains constant, resulting in the rapid death of the surviving bacteria. The only way to protect Cat(-) mutant cells against high exogenous *H*_2_ *O*_2_ concentrations is to add the wild type to the medium. This experiment has been reported by Ma and Eaton ([20]). This point will be examined in the following subsection.

### Consequence of defence switch in the primary scavenger

**Fig 11:**
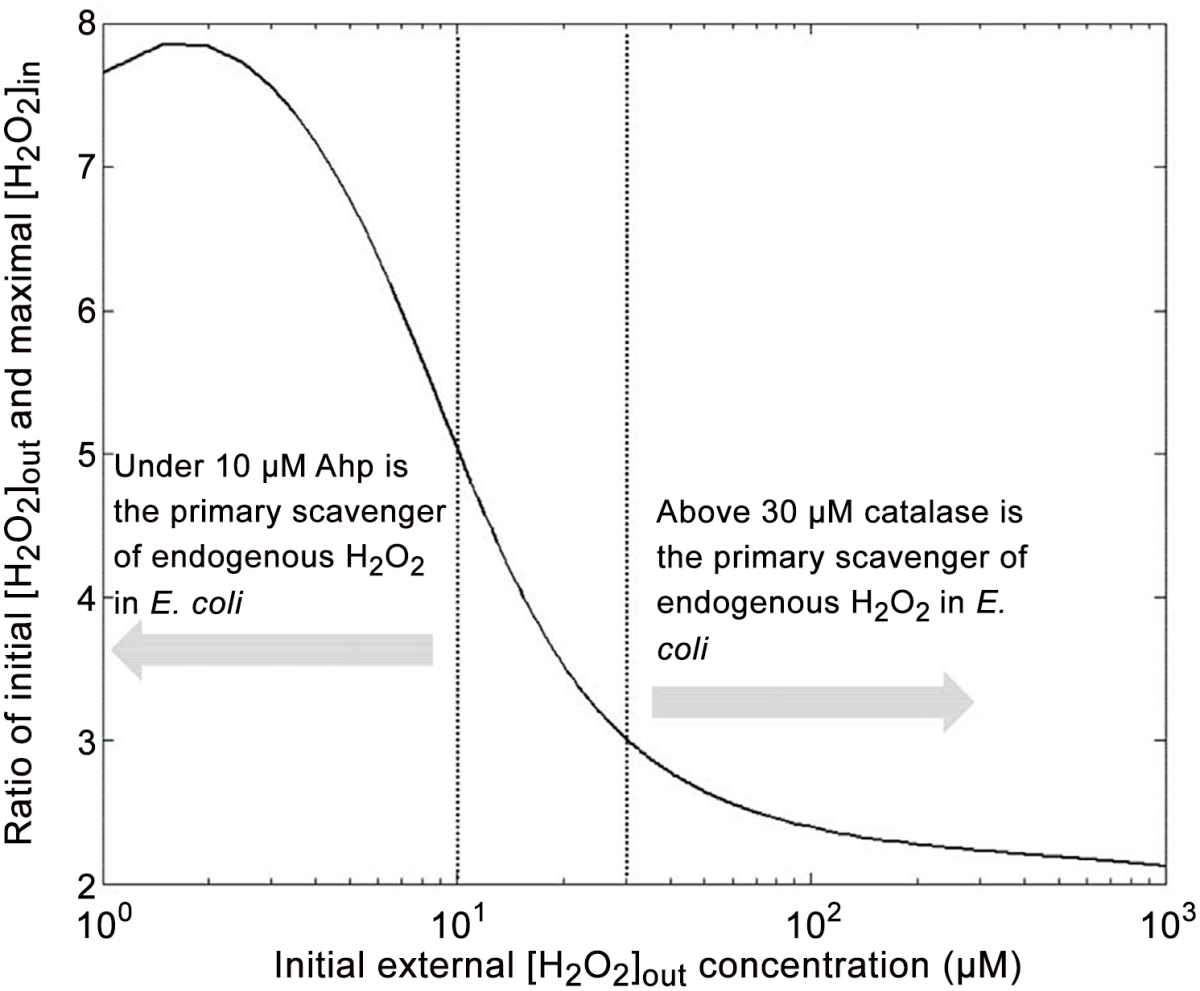
Changes in the ration between external initial *H*_2_ *O*_2_ concentration and internal maximal *H*_2_ *O*_2_ concentration in *E. coli* wild type. The numerical solution presented in this graph was running according to the whole model without approximation.

**Fig 12:**
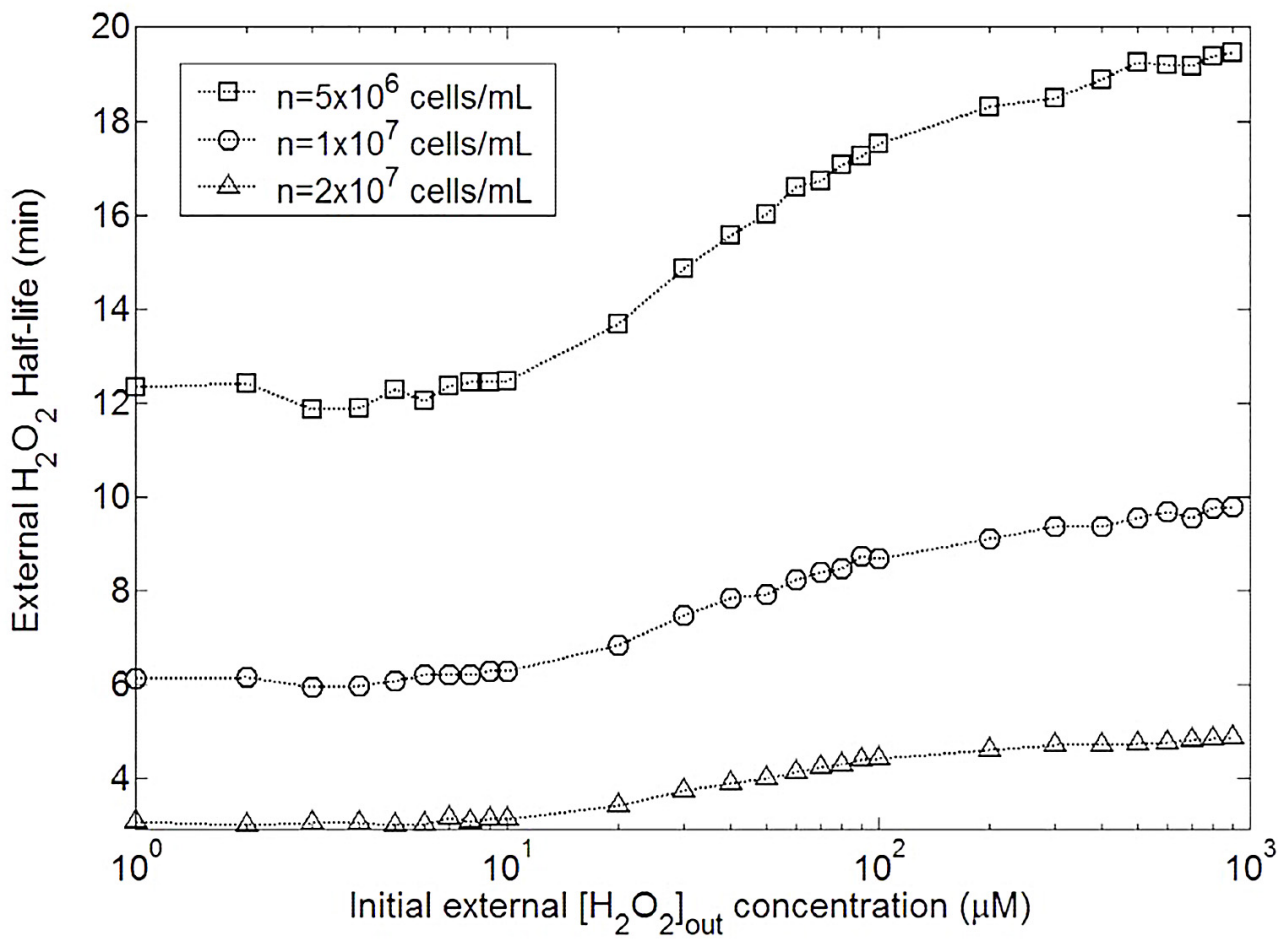
Changes in external *H*_2_ *O*_2_ half-life with different initial external *H*_2_ *O*_2_ concentrations and 3 different cell densities in *E. coli* wild type. The numerical solution presented in this graph was running according to the whole model without approximation.

Fig 11 shows that increasing exogenous *H*_2_ *O*_2_ concentration involves the switch between the two primary scavengers. This switch has already been reported by Seaver and Imlay ([5]), but we show here another consequence. Actually this switch also triggers a change in the maximal internal *H*_2_ *O*_2_ concentration viewed by cell. We also find that this maximal internal concentration does not depend on the cell density. Nevertheless the temporal internal or external *H*_2_ *O*_2_ decrease strongly depends on cell density (as previously reported in Figs 7 and 8 or in the previous subsection). The switch between the two scavengers also occurs in Fig 12, actually it shows that *H*_2_ *O*_2_ half-life increases when shifting from Ahp to Cat while exogenous *H*_2_ *O*_2_ increases.

**Fig 13:**
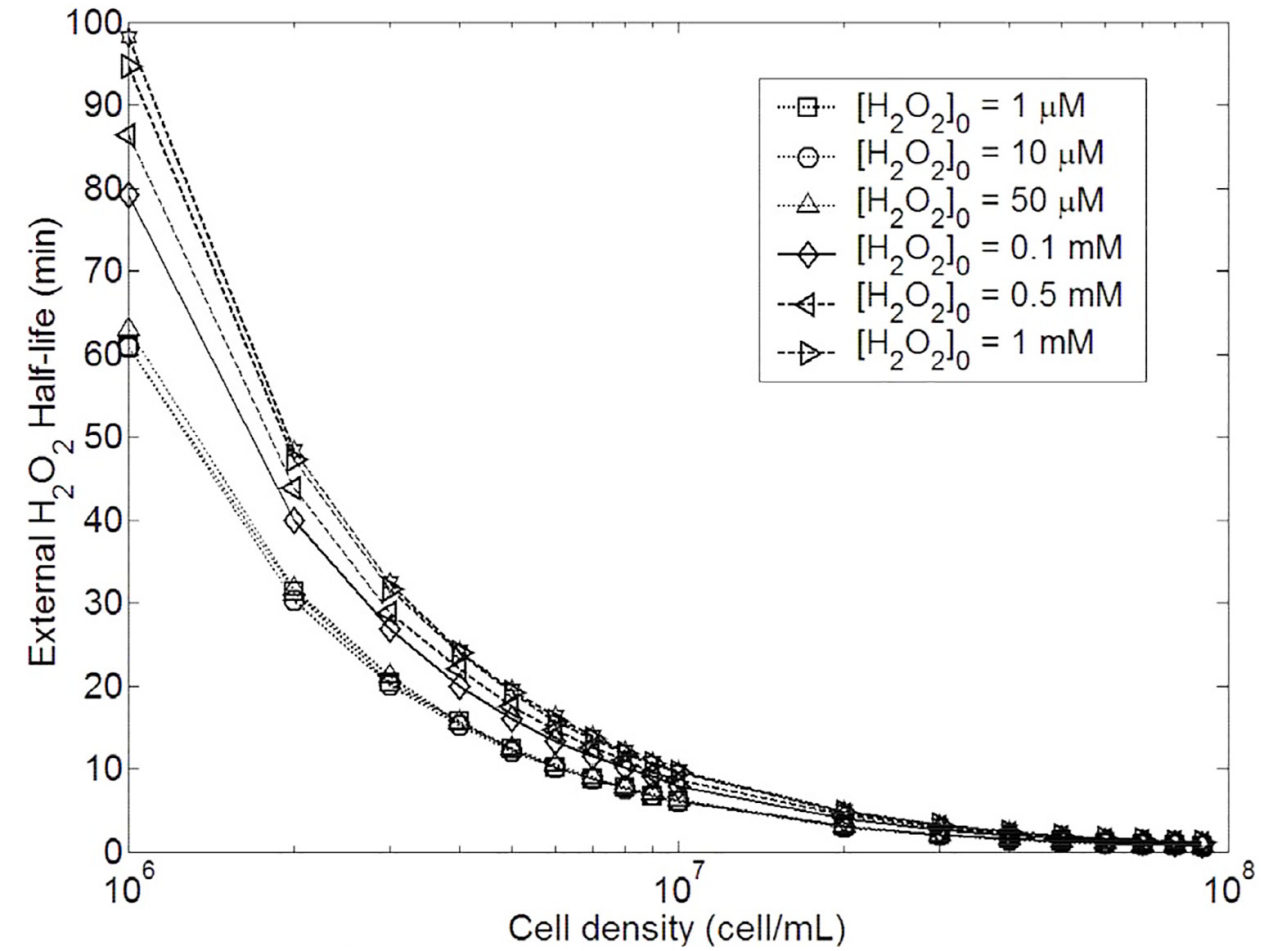
Changes in external *H*_2_ *O*_2_ half-life with cell densities and with 7 different initial external *H*_2_ *O*_2_ concentrations in *E. coli* wild type. The numerical solution presented in this graph was running according to the whole model without approximation.

This switch involves non-linear behaviour in half-life external *H*_2_ *O*_2_ dependence. Once again, Ahp seems to be more efficient but it only concerns external *H*_2_ *O*_2_ concentration under 10 *μ*M. Above 30 *μ*M, Cat plays the major role. Unlike maximal internal *H*_2_ *O*_2_, half-life depends on cell density, and the more concentrated cells are, the faster medium detoxification occurs. Nevertheless, as reported in Fig 13, under 10 *μ*M (Ahp is the primary scavenger) the half-life does almost not depend on the initial exogenous *H*_2_ *O*_2_ concentration. Above 50 *μ*M, Cat is the primary scavenger and the half-life depends on the initial exogenous *H*_2_ *O*_2_ concentration.

#### Cumulative internal *H*_2_ *O*_2_ concentration, rather than maximum internal *H*_2_ *O*_2_ concentration, is involved in the *H*_2_ *O*_2_-mediated death of bacterial cells

We investigated whether the decrease in *E. coli* survival with increasing exogenous *H*_2_ *O*_2_ concentration was linked to theoretical maximum internal *H*_2_ *O*_2_ concentration or to the rate of decrease in internal *H*_2_ *O*_2_ concentration. Indeed, a steep decrease indicates the perception of a low mean internal *H*_2_ *O*_2_ concentration by the cell. We investigated this aspect by carrying out experiments in which only one of these two parameters was affected at any one time. We therefore reproduced *in silico* the experiments of Ma and Eaton on *H*_2_ *O*_2_-mediated killing by *E. coli* wild-type (Cat(+)) or Cat(-) strains alone or by cultures of *E. coli* containing similar numbers of Cat(+) and Cat(-) bacteria. Cat(-) cells from cultures of Cat(-) cells alone or from equal numbers of Cat(-) and Cat(+) cells had similar peak *H*_2_ *O*_2_ concentrations but different rates of decrease in internal *H*_2_ *O*_2_ concentration. This result led us to evaluate the involvement of these two parameters. Moreover, as these experiments were performed with diluted and concentrated cell cultures, giving similar peak *H*_2_ *O*_2_ concentrations but different rates of decrease in internal *H*_2_ *O*_2_ concentration, we also assessed the effect of these two parameters on cell death.

**Fig 14:**
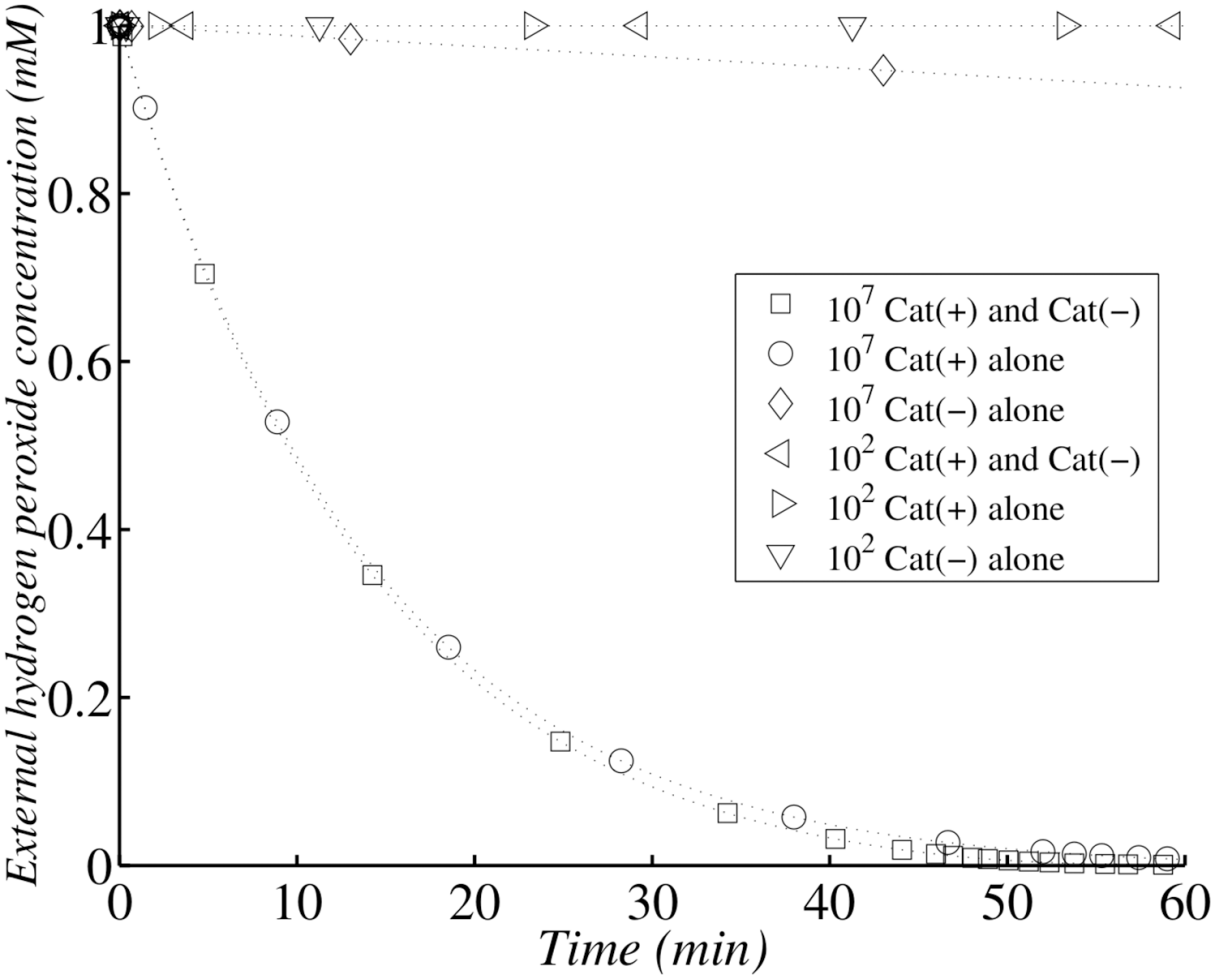
Simulation of Ma and Eaton experiment following external hydrogen peroxide concentration. Simulation of *H*_2_ *O*_2_ external concentration change with dilute (10^2^ cells per ml) Cat(-) *E. coli* alone (▿) or Cat(+) *E. coli* alone (⊳) or admixed with an equal number of Cat(+), Cat(-) *E. coli* (⊲); and with concentrated (10^7^ cells per ml) Cat(-) *E. coli* alone (◇) or Cat(+) *E. coli* alone (∘) or admixed with an equal number of Cat(+), Cat(-) *E. coli* (▫). At zero time, *H*_2_ *O*_2_ was added to a final concentration of 1.0 mM, and the bacterial suspension was then incubated at 37°C.

**Fig 15:**
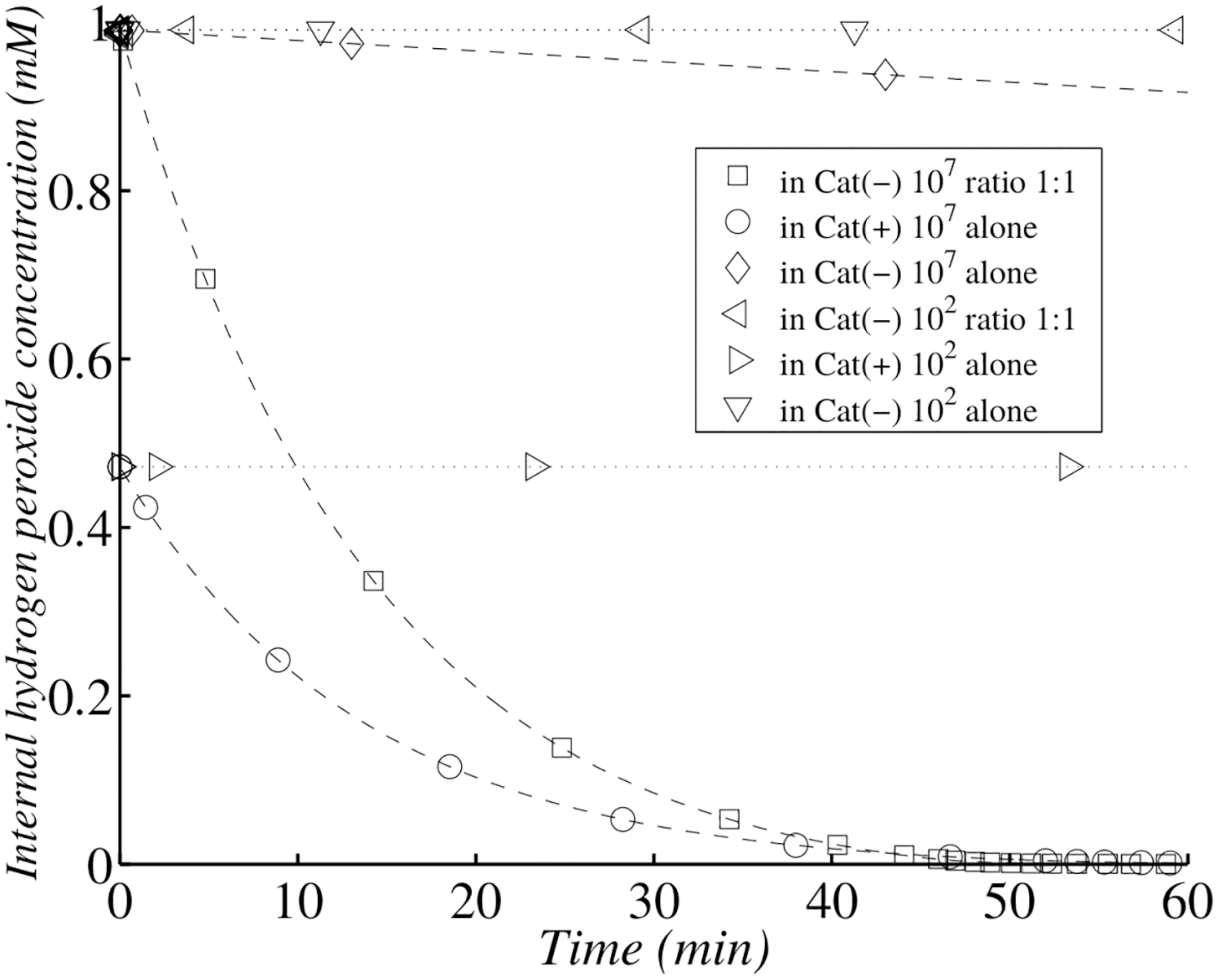
Simulation of Ma and Eaton experiment following internal hydrogen peroxide concentration. Simulation of *H*_2_ *O*_2_ internal concentration change with dilute (10^2^ cells per ml) Cat(-) *E. coli* alone (▿) or Cat(+) *E. coli* alone (⊳) or admixed with equal numbers of Cat(+), Cat(-) *E. coli* (⊲); and with concentrated (10^7^ cells per ml) Cat(-) *E. coli* alone (◇) or Cat(+) *E. coli* alone (∘) or admixed with equal numbers of Cat(+), Cat(-) *E. coli* (▫). At zero time, *H*_2_ *O*_2_ was added to a final concentration of 1.0 mM, and the bacterial suspension was then incubated at 37°C.

Simulations were performed with a dilute cell suspension (5 × 10^2^ cells ml^−1^, Figs 14 and 15) and a higher density of cells (10^7^ cells ml^−1^). Dilute populations of Cat(-) cells were unable to decrease exogenous *H*_2_ *O*_2_ concentration. Dilute populations of Cat(+) cells were also unable to detoxify the medium (Fig 14), whereas the dense population of Cat(+) cells halved exogenous *H*_2_ *O*_2_ concentration within 10 minutes (Fig 14). In a Cat(-) mutant, the maximum internal concentration of *H*_2_ *O*_2_ was only 2.5 times higher than that in Cat(+) cells, but survival rates were similar for dilute populations of both Cat(-) and Cat(+) cells ([20]). As a conclusion, the maximum internal concentration of *H*_2_ *O*_2_ is not a biological significant factor determining survival rate. Each single cell of the separate Cat(-) and Cat(+) populations had a maximum internal *H*_2_ *O*_2_ concentration of about the same magnitude, but only cells from the high-density populations survived in the Eaton experiments. Survival rate was always high when medium detoxification was activated rapidly by a dense Cat(+) cell population. Thus, even Cat(-) *E. coli* can survive if they are mixed with Cat(+) cells able to detoxify the medium. We conclude that *H*_2_ *O*_2_ scavengers do not protect individual cells against bulk-phase *H*_2_ *O*_2_, because the maximum internal concentration of *H*_2_ *O*_2_ did not differ significantly between Cat(-) and Cat(+) cells. The major difference between these two types of cells concerned the rate of decrease in exogenous *H*_2_ *O*_2_ concentration and, consequently, the rate of decrease in internal *H*_2_ *O*_2_ concentration (Fig 15). We conclude that mean internal *H*_2_ *O*_2_ concentration has a significant impact on bacterial survival, whereas maximum internal *H*_2_ *O*_2_ concentration does not. So *H*_2_ *O*_2_ action can be compared to that of the radiative exposure. This means of action is the opposite of the one generally observed for drugs. For instance, the maximum amount of paracetamol for adults is 4 grams per day with a regular intake of 0.5 gram over 3 days, but a single intake of 10 grams can lead to liver failure ([21]).

## Conclusions

### In the absence of exogenous stress

An analysis of the most significant kinetic reactions confirmed that steady-state internal concentration *H*_2_ *O*_2_ results from the balance between its production and a combination of Ahp degradation (78%), Cat degradation (12%) and membrane permeability (10%)

### With exogenous *H*_2_ *O*_2_ stress

#### Prediction of *H*_2_ *O*_2_ levels

Under conditions of exogenous *H*_2_ *O*_2_ stress, *H*_2_ *O*_2_ elimination is dependent on cell density. However, nothing is currently known about internal *H*_2_ *O*_2_ concentration during *H*_2_ *O*_2_ exposure. Under these conditions, internal *H*_2_ *O*_2_ concentration results mostly from influx due to diffusion across the cell membrane, because endogenous production is negligible. Moreover, the rate of diffusion into the cell is governed by membrane permeability. The internal concentration of *H*_2_ *O*_2_ must therefore be lower than the exogenous *H*_2_ *O*_2_ concentration. Consequently, exogenous *H*_2_ *O*_2_ stress leads to an increase in internal *H*_2_ *O*_2_ concentration until a maximum is reached. This peak is followed by a decrease in *H*_2_ *O*_2_ concentration, due to elimination by the cells. We aimed to identify the most significant parameters (kinetic constants and cell concentrations) accounting for the maximum internal *H*_2_ *O*_2_ concentration value reached and for the characteristic time points (time required to reach half the nearest steady–state concentration) during increases and decreases in internal *H*_2_ *O*_2_ concentration.

Surprisingly, based on our model, the maximal internal *H*_2_ *O*_2_ concentrations reached in individual cells was not dependent on cell density, suggesting that there is no population protection effect. This maximum, which is reached in a few milliseconds, and its characteristic timing, are dependent solely on exogenous *H*_2_ *O*_2_ concentration and the three routes of elimination of this radical (membrane permeability, Ahp and Cat scavenging).

For estimation of the maximal internal *H*_2_ *O*_2_ concentration, we needed to distinguish internal *H*_2_ *O*_2_ concentrations for which Ahp activity predominated from those for which Cat activity predominated. For initial exogenous *H*_2_ *O*_2_ concentrations below 10 *μ*M, the maximal internal *H*_2_ *O*_2_ concentration was defined by the balance between the exogenous *H*_2_ *O*_2_ diffusion rate and the three routes of elimination. In these conditions, Ahp was responsible for about 78% of all the *H*_2_ *O*_2_ eliminated. The peak internal *H*_2_ *O*_2_ concentration was almost one tenth the concomitant exogenous *H*_2_ *O*_2_ concentration. At initial exogenous *H*_2_ *O*_2_ concentrations of more than 30 *μ*M, the peak internal *H*_2_ *O*_2_ concentration was defined by the balance between the exogenous *H*_2_ *O*_2_ diffusion rate and the possible elimination routes (Ahp activity being negligible due to saturation). Thus, peak *H*_2_ *O*_2_ concentrations are determined not only by Cat activity (55%), but also by membrane permeability (45%). Surprisingly, at the peak internal *H*_2_ *O*_2_ concentration sensed by each cell, limited membrane permeability served as a passive defence against *H*_2_ *O*_2_, to a similar extent to Cat. In these conditions, internal *H*_2_ *O*_2_ concentration was only half the concomitant exogenous *H*_2_ *O*_2_ concentration.

We then showed that the rate of decrease in internal *H*_2_ *O*_2_ concentration and its characteristic timing were dependent principally on cell density and membrane permeability. This decrease was mediated not only by enzyme activity, but also by *H*_2_ *O*_2_ transport from the extracellular to the intracellular medium. The global kinetics of the decrease in internal *H*_2_ *O*_2_ concentration was determined by the slowest step in the process, diffusion across the membrane, which was limited by cell membrane permeability. Finally, similar conclusions were reported for exogenous *H*_2_ *O*_2_ concentration. The Imlay group has shown that the elimination rate for exogenous *H*_2_ *O*_2_ is much lower in intact cells than in cell extracts, indicating that diffusion across the cell membrane is the limiting process. This observation is consistent with what is known about the most significant kinetic parameters, including the major role played by the cell membrane. Indeed, diffusion across the cell membrane involves the bridging of a gap between internal and extracellular concentrations. This gap provides protection against the oxidizing extracellular medium, but it also decreases the efficiency with which *E. coli* can decrease the *H*_2_ *O*_2_ concentration of the extracellular medium (Fig 9). The kinetics of extracellular decomposition is almost exclusively diffusion-dependent and, therefore, very slow. As expected, the rate of *H*_2_ *O*_2_ disappearance (intra or extracellular) was greater at higher cell densities.

Instead of conducting real-world experiments, using simulations is generally cheaper, safer and sometimes more ethical. Simulations can also be conducted faster than experiments in real time. For instance, at the University of Pittsburgh School of Pharmacy, high-fidelity patient simulators are used in addition to therapeutics ([22]). Of course simulations have to be confronted with real experiments to test their robustness and to be improved. Our model is one step in a global modelling of the *E. coli* ROS dynamic.

“Remember that all models are wrong; the practical question is how wrong do they have to be to not be useful.”

— Box and Draper, Empirical Model-Building, p. 74

## Supporting Information

S1 File Superoxide kinetic and evolution. (PDF)
