# Supplementary material for: Hydrogen Peroxide Induced Cell Death: The Major Defences Relative Roles and Consequences in E. coli
Source: PLoS One. 2016 Aug 5;11(8):e0159706. doi: 10.1371/journal.pone.0159706 (PMC4975445; doi:10.1371/journal.pone.0159706)
Supplement: S1 File — (PDF) [file pone.0159706.s001.pdf]

Supporting Information

Superoxide kinetics

Superoxide is involved in the following kinetically significant reactions:
Its production :

$$metabolism\ production \xrightarrow{k_1} O_2^{\bullet -}$$

Its tree ways of dismutation (by SOD, by GSH and spontaneously)

$$O_2^{\bullet -} + H^+ \xrightarrow[SOD]{k_2} \frac{1}{2}H_2O_2 + \frac{1}{2}O_2$$

$$O_2^{\bullet -} + GSH + H^+ \xrightarrow{k_{GSH}} \frac{1}{2}GSSG + H_2O_2$$

$$2O_2^{\bullet -} + 2H^+ \xrightarrow{k_{sp}} H_2O_2 + O_2$$

Those reactions lead to the following ordinary differential equation (ODE) coming from the balance between production and consumption:

$$\begin{aligned} \frac{\mathrm{d}\left[O_2^{\bullet -}\right]}{\mathrm{d}t} = & \ k_1 \\ & -k_2\left[SOD\right]\left[O_2^{\bullet -}\right] - k_{GSH}\left[GSH\right]\left[O_2^{\bullet -}\right] - 2k_{sp}\left[O_2^{\bullet -}\right]^2 \end{aligned}$$

The kinetic constants used in this work are gathered in the table 1 (concerning superoxide) according to Imlay and Fridovich (1991).

**Constants**	**Value**
k_1	5.7×10^{-6} mol·L^{-1}·s^{-1}
$k_2[SOD]$	2.8×10^{4} s^{-1}
k_2	1.5×10^{9} mol^{-1}·L·s^{-1}
$k_{GSH}[GSH]$	1.3×10^{1} s^{-1}
k_{GSH}	2.6×10^{3} mol^{-1}·L·s^{-1}
k_{sp}	5.0×10^{5} mol^{-1}·L·s^{-1}

Table 1: Kinetic constants used to describe superoxide evolution.

Superoxide evolution

Without exogenous stress

In the wild-type strain

In the wild-type strain, the resolution of $O_2^{\bullet-}$ concentration gives :

$$\left[O_2^{\bullet-}\right] \approx \left[O_2^{\bullet-}\right]_\infty \left(1 - e^{-k_2[SOD]t}\right)$$

indeed GSH and spontaneous dismutation are negligible because $k_{sp} \ll k_{GSH}\,[GSH] \ll k_2\,[SOD]\,the.$ $O_2^{\bullet-}$ concentration rapidly reached its steady-state value, in less than 1 ms, actually the characteristic time is given by $\frac{1}{k_2[SOD]}$. This time, which was dependent solely on *SOD* concentration and the *SOD* catalytic degradation rate k_2, corresponds to the characteristic time require for the re-establishment of equilibrium.

If we assume that *SOD* concentration and $O_2^{\bullet-}$ production change over time, and that their equilibrium time values are probably significantly shorter than 1 ms, then the steady-state value of $O_2^{\bullet-}$ concentration is always reached but depends purely on $O_2^{\bullet-}$ production rate and *SOD* catalytic degradation rate (k_2), which are time-dependent.

For each time, we can write that the $O_2^{\bullet-}$ concentration is $\left[O_2^{\bullet-}\right](t) = \frac{k_1(t)}{k_2[SOD](t)}$. For example, with published values (Imlay and Fridovich, 1991), $O_2^{\bullet-}$ concentration is 2.1×10^{-10} M : this value fits the Imlay prediction well, and it corresponds to the equilibrium between $O_2^{\bullet-}$ production (parameter k_1) and the rate of scavenging of this radical by *SOD*.

We confirmed this approximation, by comparing the analytical solution (▲) with the numerical solution (■) in figure 1. Indeed this comparison help us to check whether the suggested approximations used to find an analytical expression are valid.

In a wild strain, the two major reaction involving superoxide are its production and its consumption by SOD.

In a SOD(-) mutant

In a *SOD* (-) mutant, changes in $O_2^{\bullet-}$ concentration were explained principally by the following differential equation :

$$\frac{\mathrm{d}\left[O_2^{\bullet-}\right]}{\mathrm{d}t} = k_1 - k_{GSH}\,[GSH]\left[O_2^{\bullet-}\right]$$

The resolution of $O_2^{\bullet-}$ concentration gave: $\left[O_2^{\bullet-}\right] \approx \left[O_2^{\bullet-}\right]_\infty \left(1 - e^{-k_{GSH}[GSH]t}\right)$, which rapidly reached its steady-state value, in less than 0.5 s. Actually the characteristic time is $\frac{1}{k_{GSH}[GSH]}$. This time, which was dependent only on *GSH* concentration and the *GSH* catalytic degradation rate k_{GSH}, corresponds to the characteristic time required for the re-establishment of equilibrium.

If we assume that *GSH* concentration and $O_2^{\bullet-}$ production can change over time, and that their equilibrium time values are probably significantly shorter than 0.5 s, then the steady state value of $O_2^{\bullet-}$ concentration is always reached but is dependent purely on $O_2^{\bullet-}$ production rate and the *GSH* catalytic degradation rate k_{GSH}, which are time-dependent.

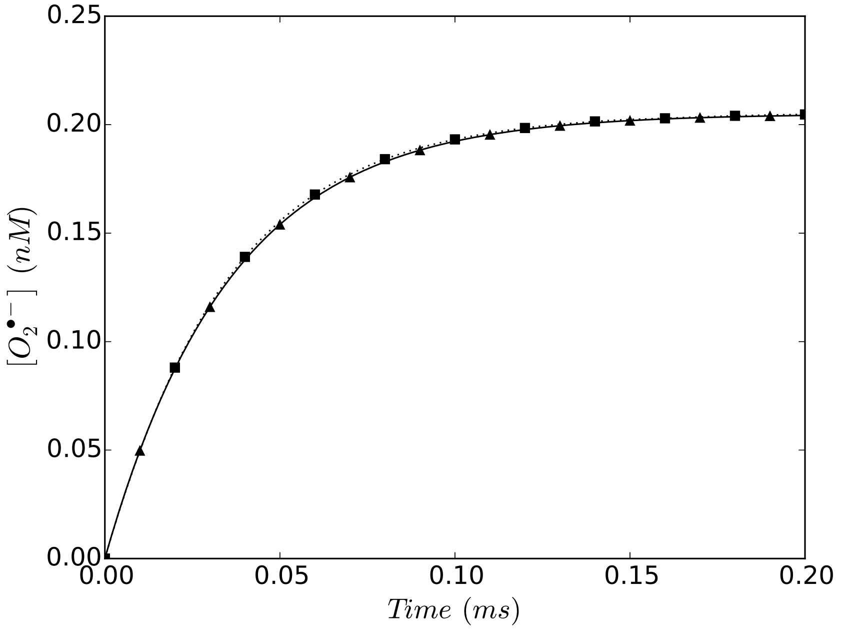

Figure 1: Change in $O_2^{\bullet -}$ concentration in the *E. coli* wild-type strain. (▲) corresponds to the analytical solution according to the simplified system and (■) corresponds to whole model solved with numerical methods. Cell density has no influence on the pattern of superoxide concentration evolution.

——————————————————Fig1

For each time point, we can write that $O_2^{\bullet -}$ concentration is $\left[O_2^{\bullet -}\right](t) = \frac{k_1(t)}{k_{GSH}[GSH](t)}$. For example, with published values (Imlay and Fridovich, 1991), $O_2^{\bullet -}$ concentration is 4.4×10^{-7} M. This value is consistent with the predictions of Imlay and corresponds to the equilibrium between $O_2^{\bullet -}$ production (parameter k_1) and the rate of scavenging of this radical by *GSH*.

For confirmation of this approximation, we compared (figure 2) the analytical solution (▲) with the numerical one (■):

In a *SOD*(-) *GSH*(-) mutant

In a *SOD*(-) *GSH*(-) mutant, the change in $O_2^{\bullet -}$ concentration can be explained principally by the following differential equation according to the spontaneous dismutation:

$$\frac{\mathrm{d}\left[O_2^{\bullet -}\right]}{\mathrm{d}t} = k_1 - 2k_{sp}\left[O_2^{\bullet -}\right]^2$$

$O_2^{\bullet -}$ concentration obeys the function :

$$\left[O_2^{\bullet -}\right] = \left[O_2^{\bullet -}\right]_\infty \frac{1 - \mathrm{e}^{-t/2\tau}}{1 + \mathrm{e}^{-t/2\tau}}$$

where $\tau = \frac{1}{\sqrt{2k_1k_{sp}}}$ and $\left[O_2^{\bullet -}\right]_\infty = \sqrt{\frac{k_1}{2k_{sp}}}$

$O_2^{\bullet -}$ concentration rapidly reaches its steady-state $\left[O_2^{\bullet -}\right]_\infty$ value, in less than 1 s (τ is the characteristic time). This time, which is dependent solely on the dismutation rate k_{sp}, corresponds to the characteristic time required to re-establish equilibrium.

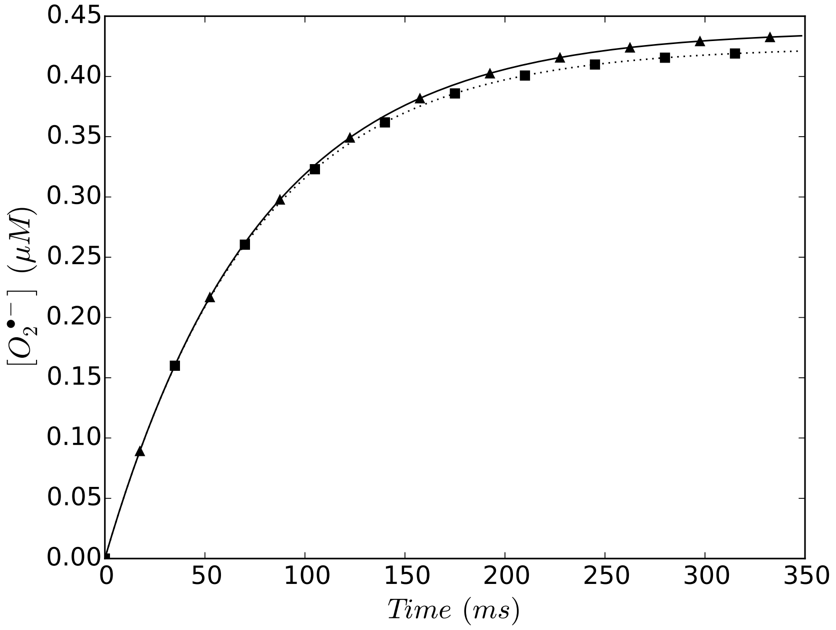

Figure 2: Changes in $O_2^{\bullet -}$ concentration in a $SOD(-)$ mutant. (▲) corresponds to the analytical solution according to the simplified system and (■) corresponds to the whole model solved with numerical methods. Cell concentration has no influence on this pattern of superoxide concentration evolution.

If we assume that $O_2^{\bullet -}$ production can change over time, and that the equilibrium time is probably significantly shorter than 1 s, then the steady-state value of $O_2^{\bullet -}$ concentration is always reached but is dependent purely on $O_2^{\bullet -}$ production rate, which is time-dependent.

For each time point, we can write that $O_2^{\bullet -}$ concentration is $\left[O_2^{\bullet -}\right](t) \approx \sqrt{\frac{k_1(t)}{2k_{sp}}}$. With published values, the $O_2^{\bullet -}$ concentration is 2.4×10^{-6}M, a value consistent with the predictions of Imlay (2001), and corresponding to the equilibrium between $O_2^{\bullet -}$ production (parameter k_1) and its spontaneous dismutation.

The analytical solution (▲) and the numerical solution (■) are the identical because they solved the same system (figure 3).

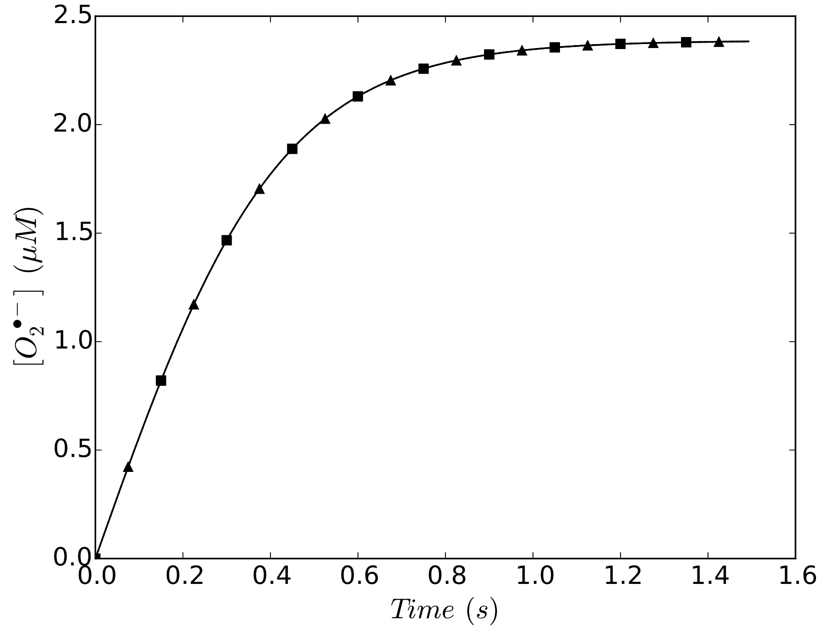

Figure 3: Change in $O_2^{\bullet -}$ concentration in $SOD(-)\,GSH(-)$ mutants. (▲) corresponds to the analytical solution according to the simplified system and (■) corresponds to the whole model with numerical methods. Cell concentration has no influence on this pattern of superoxide concentration evolution.

Superoxide summary

The table 2 summarizes the superoxide steady-state concentrations.

$[O_2^{\bullet -}]$ **(mol L^{-1})**	**In this work**	**Imlay, Fridovich**
Wild type	2.1 $\times 10^{-10}$	2.0 $\times 10^{-10}$
SOD(-) GSH(+)	4.2 $\times 10^{-7}$	4.9 $\times 10^{-7}$
SOD(-) GSH(-)	2.4 $\times 10^{-6}$	6.7 $\times 10^{-6}$

Table 2: Superoxide steady-state concentration. At steady state, the internal concentration is shown for cells in LB at 37 °C without exogenous hydrogen peroxide. In a wild-type strain, *Fe* concentration is 10 μM, and, in a SOD^{-} strain, *Fe* concentration is 80 μM (Keyer and Imlay, 1996).

References

Imlay JA, Fridovich I, Assay of metabolic superoxide production in Escherichia coli. J. Biol. Chem. 1991;266.11: 69576965.

Keyer K, Imlay JA, Superoxide accelerates DNA damage by elevating free-iron levels. Proc. Natl. Acad. Sci. U.S.A. 1996;93.24: 13635 13640.
